# Spatial and topical imbalances in biodiversity research

**DOI:** 10.1371/journal.pone.0199327

**Published:** 2018-07-05

**Authors:** Laura Tydecks, Jonathan M. Jeschke, Max Wolf, Gabriel Singer, Klement Tockner

**Affiliations:** 1 Leibniz-Institute of Freshwater Ecology and Inland Fisheries (IGB), Berlin, Germany; 2 Institute of Biology—Freie Universität Berlin, Berlin, Germany; 3 Berlin-Brandenburg Institute of Advanced Biodiversity Research (BBIB), Berlin, Germany; 4 Der Wissenschaftsfond (FWF), Vienna, Austria; Council for Scientific and Industrial Research, INDIA

## Abstract

The rapid erosion of biodiversity is among the biggest challenges human society is facing. Concurrently, major efforts are in place to quantify changes in biodiversity, to understand the consequences for ecosystem functioning and human wellbeing, and to develop sustainable management strategies. Based on comprehensive bibliometric analyses covering 134,321 publications, we report systematic spatial biases in biodiversity-related research. Research is dominated by wealthy countries, while major research deficits occur in regions with disproportionately high biodiversity as well as a high share of threatened species. Similarly, core scientists, who were assessed through their publication impact, work primarily in North America and Europe. Though they mainly exchange and collaborate across locations of these two continents, the connectivity among them has increased with time. Finally, biodiversity-related research has primarily focused on terrestrial systems, plants, and the species level, and is frequently conducted in Europe and Asia by researchers affiliated with European and North American institutions. The distinct spatial imbalances in biodiversity research, as demonstrated here, must be filled, research capacity built, particularly in the Global South, and spatially-explicit biodiversity data bases improved, curated and shared.

## Introduction

Biodiversity–from genes to ecosystems–represents the combined biological information that has accumulated over billions of years of evolution. Up to now, humanity is far from being able to determine its amount and value, and to estimate the consequences that an expected 10, 20 or even 50% decline of biodiversity may have for sustaining vital ecosystems and human wellbeing. Indeed, biodiversity information across all levels of biological organization is fundamental in understanding how the Earth system functions, and how it interacts with human activities [1]. To stop, and potentially reverse, the rapid erosion of biodiversity, major efforts are in place, guided by the Intergovernmental Platform on Biodiversity and Ecosystem Services (IPBES), the UN Sustainable Development Goals, the EU Biodiversity Strategy (EC 2011), and various EU directives (i.e. Water Framework Directive, Habitats Directive). Furthermore, conservation efforts must be accompanied by education and capacity building.

Biodiversity research has a distinct spatial component and, therefore, may be exposed to geographic biases such as an underrepresentation of research activities in the Global South. Similar biases have been discovered in research on climate change [2], invasive species [3,4], and in part biodiversity (e.g. [5,6]). In the Global South, for example, biodiversity is understudied and little protected due to a lack of awareness and funding alike [7]. Indeed, entire regions suffer from a lack of research capacity, and existing research is weakly integrated with local knowledge. Yet the same regions represent key target areas for conservation and the sustainable use of biodiversity-related resources.

Current efforts to compile information on biodiversity include the establishment of data bases such as the Global Biodiversity Information Facility, summarizing species occurrence data (GBIF; [8]), or GenBank, focusing on genetic diversity. Such data bases could help discover “black spots” in biodiversity knowledge, i.e. countries and regions of limited knowledge, and facilitate conservation efforts [9]. However, these laudable efforts notably suffer from spatial and topical research deficits themselves. For example, species occurrence data are spatially co-located with research centres [10], data are prone to spatial and taxonomic errors [11], and the ecosystem component of biodiversity is particularly ignored. To better support decision making and conservation planning, biodiversity data bases need a balanced spatial and topical coverage, which requires major efforts in addressing existing and emerging biodiversity research deficits.

In the present study, we assume that biodiversity is best studied (i) where it is actually located, (ii) at all levels of ecological organization (i.e., genetic, species, ecosystem level), and (iii) equally distributed across the phylogenetic branches of the tree of life. While this seems to be a reasonable assumption, other approaches are conceivable, too. For example, some taxonomic groups are easier to study due to their accessibility and thus a focus on such proxy groups–in combination with an extrapolation to other taxa–can be cost-effective [12]. Alternatively, one could focus on keystone species or ecosystems where the benefits are most likely higher through an increased system understanding [13]. With regard to our assumption, we ask whether biodiversity research has been doing it right over the past decades. More specifically: Have we put our efforts at the right place? Have we been looking at the right components?

Based on comprehensive bibliometric analyses, we investigated the spatial (i.e. geographical) context and topical focus of all 134,321 biodiversity-related publications included in Web of Science (WoS). To cover this bibliographic information, we used automatic search algorithms–in contrast to previous bibliometric studies, which focused on a small subset of biodiversity-related studies. Our investigation includes analyses of (a) research flows among countries and regions contributing to science and those rich in biodiversity resources, (b) the spatial distribution and connectivity of identified core scientists in biodiversity research, (c) (spatial) research deficits with regard to threatened species and ecoregions, and (d) spatial-topical research foci.

We expected a strong spatial bias at the global scale because biodiversity research is most likely dominated by scientists based in the Global North. Accordingly, we expected spatial disagreement between biodiversity hot spots and locations of biodiversity-related research efforts. Furthermore, we expected that most biodiversity-related research has been conducted in terrestrial systems, with a focus on plants, and at the species level. Yet such topical biases are likely not spatially uniform at the global scale. Based on our findings, we outline and discuss consequences of (un)balanced biodiversity research activities for the understanding and tackling of current and future changes of biodiversity *sensu lato*.

## Methods

### Search strategy and setup of data base for bibliographic information

In the present context, biodiversity-related research includes research on the diversity of genes, species, and ecosystems in all realms of life as well as associated topics where biodiversity matters, (e.g., prevalence or spread of diseases, food provision).

In a first step, we conducted a Web of Science (WoS) Core Collection search using the term ‘biodiversity’. Out of 76,594 publications, 100 articles were randomly selected and titles and abstracts were read to get a first overview of biodiversity-related terms. In a next step, these terms were discussed among the authors and selected for a comprehensive search: biodiversity, biological diversity, species richness, species evenness, genetic diversity, species diversity, ecosystem diversity, alpha diversity, beta diversity, gamma diversity, taxonomic diversity, phylogenetic diversity, behavio(u)ral diversity, functional diversity.

In a second step, we used these terms and searched the WoS again for all articles, published in English from 1945 to 2014, and downloaded the bibliometric information including title, abstract, author, affiliation, country of affiliation, publication year, and the number of times an article was cited (download date: May 12, 2015). In total, 134,321 biodiversity-related and peer-reviewed publications were compiled in a SQL-data base for further analyses (Data available from the Dryad Digital Repository: doi:10.5061/dryad.q7mk04m). We did not restrict the WoS search to specific journals in order to guarantee a broad coverage of biodiversity-related research, considering publication dynamics during the past 60 years. Thus, the data base includes studies that otherwise might have been overlooked. However, WoS does not cover grey literature (https://clarivate.libguides.com/woscc), and we did not consider other languages than English. Hence, the actual number of studies related to biodiversity is higher than reflected in our WoS search strategy. Nonetheless, it provides a highly representative sample of scientific research, appropriate to detect and understand imbalances related to biodiversity research.

### Spatial distribution of biodiversity research

We identified study sites of publications at the country level and crossed this information with the country of an author's affiliation as well as with geographical biodiversity- and economy-related data. To detect locations of study sites, all publication titles and abstracts were automatically searched for country names, using R version 3.2.2 [14]. For large countries (i.e. more than 2.5 million km^2^; i.e. Russia, China, USA, Canada, Brazil, Australia, India, Argentina and Kazakhstan), we additionally searched for predefined province names. Study sites at the country and province level were identified for a total of 49,932 publications (37.2% of all studies). As the country of the study site was not necessarily identical with the country of the author affiliation, we also extracted geographical information on author affiliation. This allowed quantifying country-specific contributions to biodiversity research. Errors in this information, such as typos, were manually corrected.

Data extracted from the bibliographic data base, such as the number of biodiversity-related publications per country, were analysed in conjunction with several quantifiers of biodiversity that were available at country level: The number of threatened species (IUCN Red List of Threatened Species; [15]), the number of ecoregions (WWF List of Ecoregions; [16]) and the proportion of protected areas [17]. Similar to data on diversity of amphibians (AmphibiaWeb [18]) and birds (BirdLife International [19]), these biodiversity quantifiers may serve as proxies for overall biodiversity. The number of ecoregions was significantly correlated with the number of amphibians (r = 0.75, p < 0.001) and birds (r = 0.81, p < 0.001) (S3 Table). Hence, the number of ecoregions was used as a surrogate for biodiversity at the country level. The proportion of protected areas is further testifying conservation efforts. Last, the bibliometric information of a country was correlated with its economic performance (Gross Domestic Product (GDP) (http://unstats.un.org).

To visualize spatially resolved data, such as the ratio between the number of biodiversity-related studies and other quantities (i.e. number of ecoregions or threatened species) for countries and provinces, we calculated cartograms (90 iterations) using QGIS version 2.12.0 [20]. For these analyses, only publications at species or ecosystem level were considered. Also, these analyses were limited to amphibians (2372 publications) and birds (8325 publications), for which biodiversity data were readily available at reasonable geographical resolutions (see above). To quantify research flows from the continental regions where authors were affiliated to the regions where studies were conducted [21], we used a Sankey diagram. To simplify the presentation, we aggregated the country-specific data to continental regions following the United Nations classification (http://unstats.un.org).

Finally, we identified “core scientists” and their institutional affiliation(s) from the bibliographic data base. For each five-year period, from 1945 to 2014, the twenty most frequently cited scientists, based on the total number of citations, were classified as “core scientists”. Scientists were excluded if the domain of their research was not biodiversity or if an author was solely listed as co-author of one highly cited publication, without any further documented research output in the dataset. For the periods 1980–84 and 2010–14, a sensitivity analysis was conducted by gathering information on ten additional core scientists (S2 Table). Our approach for identifying “core scientists” is relatively straightforward, yet other possibilities for creating lists of influential scientists can be imagined as well, e.g. by considering higher weights for first or senior authors as compared to other co-authors, or by using the h-index. The list of identified “core scientists” was not meant to be exhaustive, hence researchers not on the list could have been very influential as well.

To identify potential centers of biodiversity research, we collected information about the affiliation of each core scientist at the time of the doctoral degree and the last confirmed (or current) affiliation. In total, we gathered detailed information on 156 core scientists, but could not obtain the required information for another 19 core scientists. The activities of several core scientists spanned more than one five-year period. Furthermore, the gender of a total of 154 core scientists was recorded (based on online material of institutions and organizations and the CVs of the core scientists). In order to visualize the degree of connectivity among core scientists, we conducted a network analysis using Gephi [22]. Modularity clusters, betweenness centrality, as well as the average degree and the mean weighted degree of the nodes (i.e. core scientists) were calculated for the time periods 1980–94, 2000–14 and 1945–2014. The modularity clusters summarize scientists that are more densely connected (through publications) among each other than to the rest of the scientists. The betweenness centrality represents the shortest path from all core scientists to all other core scientists, identifying central core scientists. The degree of a node represents the number of relations it has, i.e. how many papers one core scientist published with other core scientists.

### Spatial and topical distribution of biodiversity research

To identify topical foci and their geographical distribution, we allocated publications to the level of ecological organization (genetic, phylogenetic, species, ecosystem), the taxonomic/functional group (plants and algae, invertebrates, vertebrates, bacteria, fungi, viruses, parasites), and the research domain (terrestrial, freshwater, marine). Relevant terms were identified from randomly selected subsets of 50 publications from each decade between 1945 and 2014. Furthermore, the taxonomic/functional groups of the kingdoms Animalia, Plantae, Fungi and Virus were used as search terms (Integrated Taxonomic Information System, IT IS; http://www.itis.gov; S1 Table). Again, all publication titles and abstracts were automatically searched for those terms. To test whether the automatic allocation to research foci was consistent, the accordance with the subsets of the first two decades (2 and 13 publications, respectively) was manually checked. For other decades, 95%-confidence intervals for fractions allocated to topics were calculated for the subset of 50 publications using the binom package in R [23] and compared to fractions automatically allocated for the entire set of publications (S1–S3 Figs). Using this approach, we successfully identified the level of ecological organization for a total of 116,368 publications (86.6%), the taxonomic/functional group for 86,401 publications (64.3%), and the research domain for 96,933 publications (72.2%). To identify the spatial distribution of research foci, we linked the topical with the spatial information of the publication.

## Results

**Fig 1:**
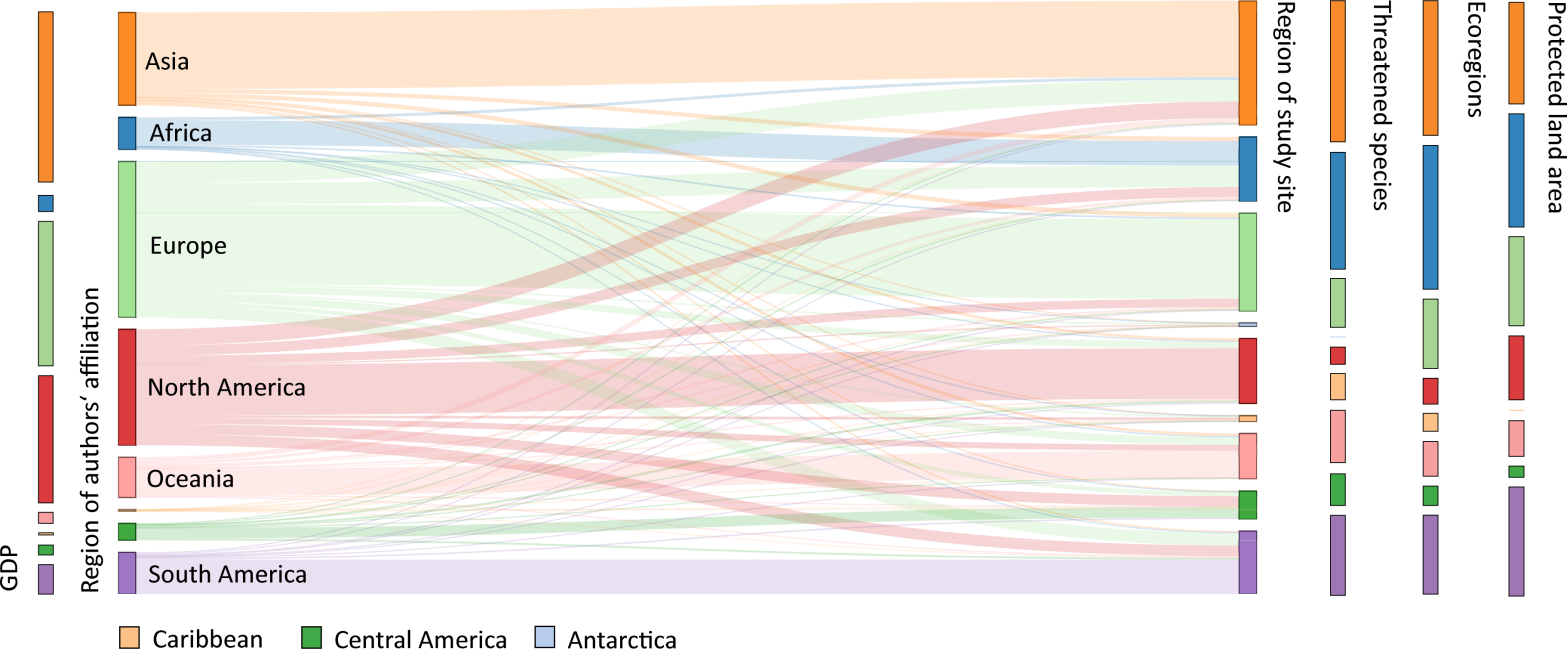
Sankey diagram quantifying research flows from the region of author affiliation to the region of research conductance. Vertical bars: GDP, the number of threatened species, the number of ecoregions and protected land surface area in each region (relative values; n = 49,932).

Research efforts (based on authors’ affiliation of biodiversity-related publications) were highest in Europe (31.2% of all publications), followed by North America (23.2%) and Asia (18.6%). When only the first-author affiliation of each publication was considered, the relative research efforts further decreased in Africa, Central America and the Caribbean (S4 Table). The majority of study sites of biodiversity-related publications were located in Asia (24.9% of all publications), Europe (19.6%) and North America (13.0%) (Fig 1). Europe and North America were the main research export regions (difference between research effort and study sites: -11.6% and -10.2%, respectively), while Asia and Africa were the main import regions (+6.3% and +5.6%, respectively) (Fig 1).

The research efforts of a region, based on authors’ affiliation, was significantly and positively correlated with the economic strength (GDP) of that region (r = 0.9, p < 0.001; Table 1). In contrast, there was no significant correlation between research effort in a region and proportion of threatened species (r = 0.2, p = 0.61), number of ecoregions (r = 0.3, p = 0.42), and total protected area (r = 0.5, p = 0.111) in that region (Table 1).

**Table 1:** Correlations of GDP, number of threatened species, number of ecoregions and total size of protected areas (each for all 8 regions) with the number of publications for each continental region based on authors’ affiliation and on study site. Significant correlations are highlighted in bold.

	Region-wide number of publications based on authors’ affiliation		Region-wide number of publications based on study site	
Regional variable	correlation coefficient	p-value	correlation coefficient	p-value
**GDP**	***0*.*9***	***< 0*.*001***	***0*.*87***	***< 0*.*01***
**Threatened species**	0.2	0.61	***0*.*72***	***< 0*.*05***
**Ecoregions**	0.31	0.42	***0*.*74***	***< 0*.*05***
**Protected area**	0.56	0.11	***0*.*82***	***< 0*.*01***

In total, biodiversity-related research primarily focused on terrestrial systems (83.3% of all publications), on plants (55.8%), and on the species level (68.3%), with a major share of authors affiliated with European (24.4% on terrestrial systems; 24.1% on plants; 23.5% at the species level) and North American (18.1%; 16.9%; 17.3%) institutions (Table 2A). When looking at study sites rather than affiliations, more than a third of studies on terrestrial systems, on plants and at the species level were conducted in Europe (14.9%; 15.4%; 14.1%), Asia (14.1%; 14.2%; 12.9%) and North America (10.4%; 10.4%; 10.2%) (Table 2B).

**Table 2:** Contributions to publications (in %) at the level of ecological organization, research domain, and taxonomic/functional group by continental region according to (A) author affiliations and (B) study sites. Percentages add up to more than 100% due to publications that cover more than one given level of ecological organization, research domain, or taxonomic group.

**A) Affiliations**	**Africa**	**Asia**	**Europe**	**North America**	**South America**	**Central America**	**Caribbean**	**Oceania**	**Antarctica**	**not allocated**	***Publications*** ***(total)***		***Publications*** ***(%)***
**Level of ecological organization**													
genetic	5.0	18.0	20.5	14.1	5.9	1.9	0.3	4.5	-	29.8	*55747*	*47*.*9*	
species	4.1	11.6	23.5	17.3	6.6	2.6	0.2	6.2	-	27.9	*79540*	*68*.*4*	
ecosystem	3.9	10.0	20.8	17.5	5.0	2.0	0.1	6.6	-	34.1	*18423*	*15*.*8*	
phylogenetic	4.4	18.5	19.9	15.0	5.4	2.0	0.5	4.6	-	29.6	*9389*	*8*.*1*	
**Taxonomic group**													
Vertebrates	4.9	10.6	21.1	22.8	7.5	2.9	0.2	8.4	-	21.6	*25419*	*29*.*4*	
Invertebrates	4.3	10.5	26.2	17.5	7.8	2.6	0.2	6.1	-	24.9	*19909*	*23*.*0*	
Plants and algae	4.8	13.3	24.1	16.9	6.1	2.6	0.2	6.2	-	25.9	*48212*	*55*.*8*	
Fungi	2.2	14.3	23.0	12.5	5.1	2.1	0.2	3.7	-	36.9	*3902*	*4*.*5*	
Virus	10.0	21.6	23.1	17.3	8.0	1.6	0.7	3.2	-	14.6	*3838*	*4*.*4*	
Bacteria	2.7	11.1	15.2	9.4	3.5	1.2	0.2	2.6	-	54.3	*7472*	*8*.*7*	
Parasites	8.9	12.5	24.4	16.7	9.6	3.1	0.1	5.9	-	18.9	*2932*	*3*.*4*	
**Research domain**													
terrestrial	4.8	12.9	24.4	18.1	6.3	2.6	0.2	6.4	-	24.4	*80749*	*83*.*3*	
freshwater	3.9	11.7	24.7	22.0	6.4	1.4	0.2	6.3	-	23.4	*17502*	*18*.*1*	
marine	3.1	11.4	22.4	18.3	8.7	3.0	0.3	8.2	-	24.7	*19044*	*19*.*7*	
**B) Study sites**	**Africa**	**Asia**	**Europe**	**North America**	**South America**	**Central America**	**Caribbean**	**Oceania**	**Antarctica**	**not allocated**	***Publications*** ***(total)***	***Publications*** ***(%)***	
**Level of ecological organization**													
genetic	6.9	19.3	11.3	6.8	6.8	2.8	0.7	4.8	0.4	40.2	*55747*	*47*.*9*	
species	6.1	12.9	14.1	10.2	7.8	3.5	0.8	6.2	0.5	38.0	*79540*	*68*.*4*	
ecosystem	5.1	10.8	12.6	10.7	5.7	2.5	0.6	5.9	0.6	45.5	*18423*	*15*.*8*	
phylogenetic	7.0	19.9	9.6	5.9	6.4	3.0	1.0	5.2	0.6	41.4	*9389*	*8*.*1*	
**Taxonomic group**													
Vertebrates	7.4	12.1	11.2	12.7	8.8	4.1	0.9	8.2	0.3	34.3	*25419*	*29*.*4*	
Invertebrates	6.5	11.8	16.1	10.3	8.9	3.8	0.9	6.7	0.4	34.6	*19909*	*23*.*0*	
Plants and algae	6.3	14.2	15.4	10.4	7.3	3.4	0.6	5.9	0.3	36.3	*48212*	*55*.*8*	
Fungi	3.0	14.7	15.3	7.3	5.8	2.7	0.5	3.5	0.9	46.4	*3902*	*4*.*5*	
Virus	11.3	22.4	12.8	7.1	8.6	2.2	1.2	3.4	0.0	31.0	*3838*	*4*.*4*	
Bacteria	3.1	11.1	9.1	5.5	3.9	1.6	0.4	2.2	1.2	61.9	*7472*	*8*.*7*	
Parasites	11.4	12.2	10.7	6.7	10.0	4.1	1.0	8.4	0.3	35.3	*2932*	*3*.*4*	
**Research domain**													
terrestrial	6.7	14.1	14.9	10.4	7.6	3.4	0.7	6.2	0.4	35.6	*80749*	*83*.*3*	
freshwater	5.3	12.2	17.4	16.0	7.4	2.0	0.6	6.7	0.6	31.9	*17502*	*18*.*1*	
marine	5.4	14.6	11.0	11.0	9.7	4.3	1.2	8.8	1.4	32.7	*19044*	*19*.*7*	

The major part of genetic and phylogenetic studies was conducted in Asia (19.3%), the major part of species (14.1%) and ecosystem studies (12.6%) in Europe. Studies on invertebrates (16.1%), plants and algae (15.4), and fungi (15.3) were mainly conducted in Europe; on viruses (22.4%), bacteria (11.1%) and parasites (12.2%) mainly in Asia, and studies on vertebrates (12.7%) mainly in North America. Terrestrial (14.9%) and freshwater studies (17.4%) were mainly conducted in Europe, marine studies (14.6%) mainly in Asia (Table 2B).

**Fig 2:**
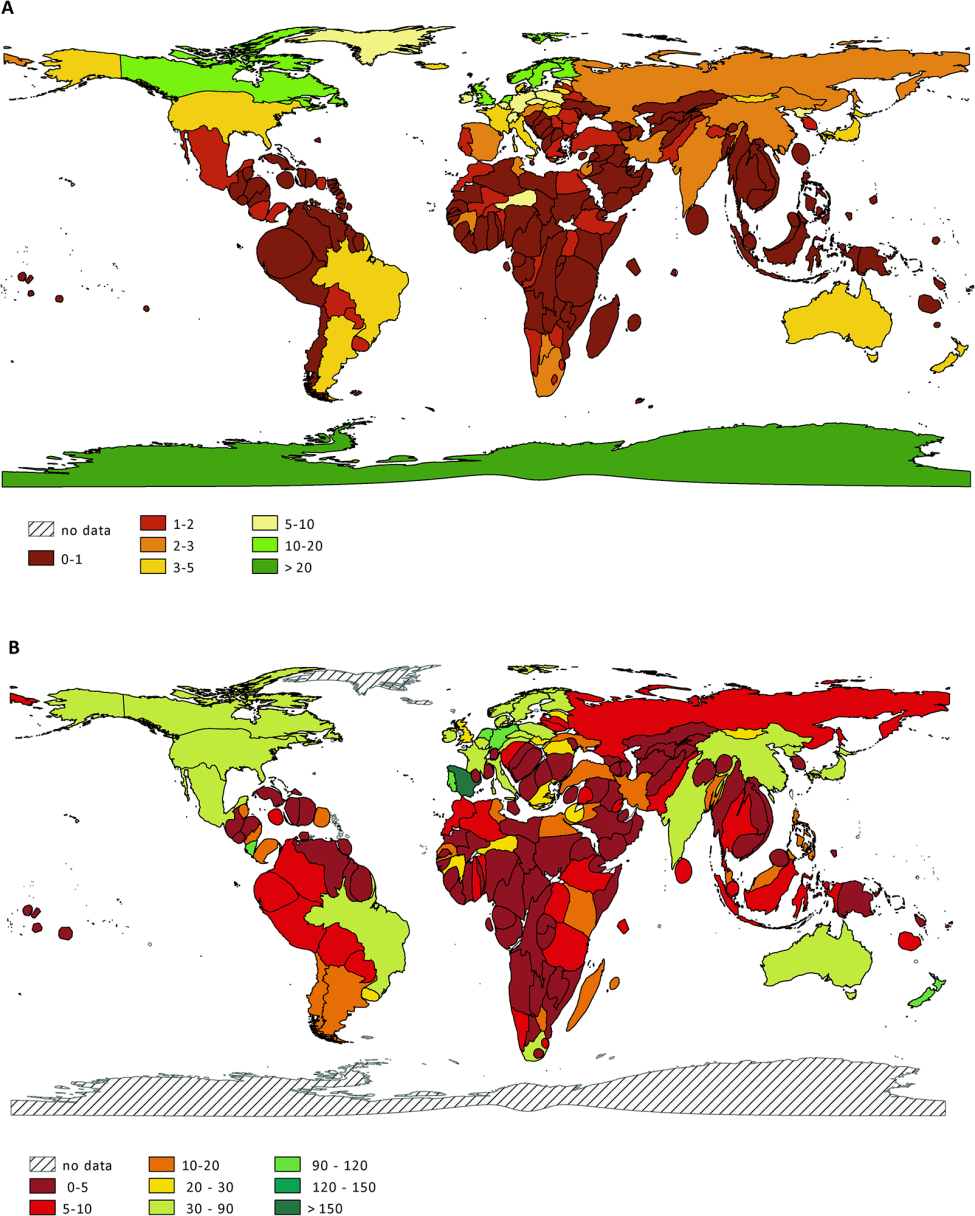
Cartograms showing ratios between biodiversity-related research effort and biodiversity quantifiers. In **(A)** the size of each country represents the number of threatened species (based on the IUCN Red List); the color represents the ratio between publication count and number of threatened species. In **(B)** the size of each country represents the number of ecoregions (based on the WWF List of Ecoregions); the color represents the ratio between publication count and number of ecoregions. In both (A) and (B), red countries have fewer studies per threatened species or per ecoregion and thus exhibit a relative biodiversity research deficit. The cartograms were generated using QGIS version 2.12.0 [20].

Focusing on individual countries, the number of studies per ecoregion (i.e. location of the study) was highest in Spain (171.5 publications per ecoregion), Portugal (112), the Netherlands (106), Germany (103.5) and Sweden (85.5); compared to very low ratios in countries in the Global South (e.g., Laos: 2.0, Papua New Guinea: 2.5, Cameroon: 3.8) (Fig 2A). Similar ratios were detected for studies on amphibians and birds (S4 Fig). Likewise, the highest number of studies per threatened species was carried out in European countries (Finland: 19.9 publications per threatened species, Sweden: 18.7, United Kingdom: 17.4, Norway: 11.6, the Netherlands: 11.5) and Canada (19.4). In contrast, the number of studies per threatened species was disproportionately low in Malaysia (0.3), Papua New Guinea (0.31), Madagascar (0.45) and Nicaragua (0.64) (Fig 2B).

**Fig 3:**
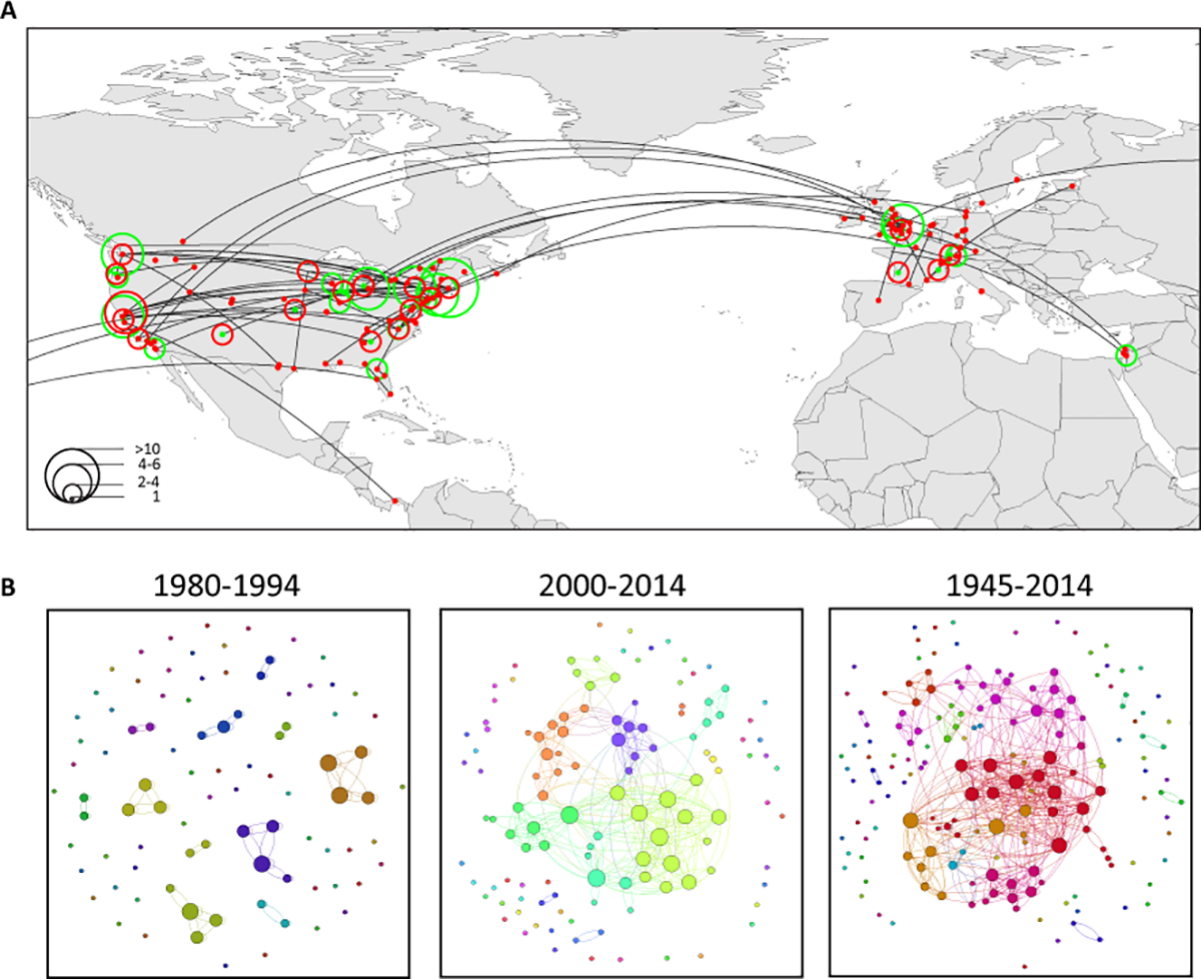
Core scientists in biodiversity research. **(A)** Global distribution of identified core scientists in biodiversity research, with PhD (green) and current (red) affiliation. The size of a circle represents the number of core scientists. The lines represent the movement of each individual scientist from the PhD location to the current affiliation. The map focuses on North America and Europe, as 142 out of 156 core scientists in biodiversity research (91%) were affiliated to institutions in these two continents. The map was generated using QGIS version 2.12.0 [20]. **(B)** Core scientists’ network during different time periods. Colors represent modularity clusters. Node size represents degree (centrality), i.e. how many publications one core scientist published with other core scientists. The network was generated using Gephi version 0.8.2 [22].

From 1945 until today, 142 out of 156 identified “core scientists” (see Methods) in biodiversity research were affiliated to institutions in North America and Europe (91%; Fig 3A, S5 Table). Nine of the top-10 institutions, based on the number of affiliated core scientists (either for their doctoral degree or their current affiliation), were located in the USA. Harvard University (15 core scientists) was followed by the University of Washington (8 core scientists). The only non-US institution in the top-10 list was the University of Cambridge, UK (7 core scientists). Movement from an institution where a core scientist received the doctoral degree to the current affiliation was mainly within and between North America and Europe. The highest net influx between doctoral degree (no core scientist) and current affiliation was found for the University of California, Davis (6 core scientists). In contrast, the highest efflux occurred from Harvard University (doctoral degree: 11 core scientists; current affiliation: 4).

Core scientists in biodiversity research were mainly affiliated with universities and colleges (241 scientists; 88.9%) (Fig 3A, S6 Table). More than 90% of the core scientists were males, and this proportion remained high over time (S7 Table).

The degree of connectivity among core scientists, expressed through joint publications, strongly increased over time (Table 3, Fig 3B–3D). The modularity clusters summarize scientists that are more densely connected (through publications) among each other than to all other scientists. While 20 years ago scientists mainly published within their modularity cluster (i.e. scientists that are densely connected), connectivity among clusters of scientists increased with time (1980–94: modularity 0.8, 2000–14: modularity 0.6; Table 3, Fig 3B–3D).

**Table 3:** Network statistics describing the collaboration among identified core scientists working on biodiversity during different time periods.

Time period	Average degree	Mean weighted degree	Modularity	Number of modularity clusters
1980–1994	1.533	4.533	0.85	71
2000–2014	3.885	36.016	0.634	63
1945–2014	3.702	33.095	0.647	87

## Discussion

Biodiversity-related research has strongly increased since the 1980s, mainly due to a growing recognition of the rapid decline of biodiversity, its pivotal value for nature and humans alike, and the subsequent ratification of the Convention on Biological Diversity (CBD). Our quantitative analyses of three biodiversity categories (i.e., level of ecological organization, research domain, taxonomic group), as well as spatially-explicit assessments of research efforts, uncovered distinct imbalances in biodiversity research, emphasizing previous findings (e.g.[6,24–26]). Notably, we analysed the most comprehensive and up-to-date dataset available so far.

Biodiversity research has focused on particular regions of the world, on terrestrial systems, on plants, and at the species level. One underlying reason for the spatial and topical imbalance may simply be the so-called Matthew principle, which explains the concentration of research on already well-studied subjects for extended periods of time [5,24,25]. Based on the present results, there is an urgent need for a more balanced, spatially and topically well-adjusted biodiversity research portfolio.

First and foremost, our global analyses show that biodiversity is not primarily investigated where it is actually located. Human resources in biodiversity research, and the related capability to disseminate knowledge, are mostly restricted to North America and Europe–regions with strong economic performance. At the same time, countries with high biodiversity, expressed by the number of ecoregions, and high proportion of threatened species remain underrepresented in research. Most of these countries are located in economically weak regions in Africa, Asia and South America. Fortunately, the number of taxonomists based in South America and Asia, where most species occur, is actually increasing [26]. Yet, the contributions to publications by researchers in developing countries, such as those on the African continent, are frequently through access to study sites and provisioning of data [27]. Study design, laboratory work, and data analyses are carried out by institutions located in the northern hemisphere [27–29], resulting in a strong dependency of biodiversity-rich regions on institutions in the Global North with respect to knowledge production, publications, and scientific reputation [29]. Hence, the knowledge and performance of scientists further increase in wealthy, rather than in biodiversity-rich and capacity-poor regions.

Cooperation in biodiversity research at the global scale is a rather recent phenomenon [30]. Increasing interdisciplinarity and at the same time specialisation of researchers, pooling of research facilities and resources to reduce costs, as well as global funding opportunities are the main reasons for a growing international collaboration [31–34]. Our analyses of co-publications, as an indicator of national and international collaboration, show that the degree of connectivity among core scientists has strongly increased over time. Notably, cooperation within and between the United States and Europe by far trumps cooperation across wider geographic and institutional borders, which may be due to the strong economic performance of both regions [31] and the general tendency of higher collaboration within geographical proximity [32].

Global initiatives, such as IPBES and GEO BON, foster collaborations and extend communication paths across geopolitical borders. Indeed, global collaboration is fundamental considering the spatial and temporal variability of biodiversity and, hence, to detect trends and to close knowledge gaps (e.g. number of species; [35]). Unfortunately, for example in ecology, most data are only accessible as interpretations through publications, while only a small fraction is directly accessible [36]. Similarly, there is a lack of long-term data on climate change in the Global South, which is considered problematic due to the potential impacts of climate change on biodiversity [37]. At the same time, biodiversity monitoring programs are challenged by incomplete taxonomic and spatial data coverages [38]. For example, global data bases such as GBIF are geographically biased: North America, Europe, and Australia are the regions where most of the data are digitized [10]. Indeed, open science and access to data bases, considering intellectual property rights and ethical aspects, are crucial in supporting a fair global knowledge exchange. At the same time, a fast increase of biodiversity data, in particular through rapidly advancing molecular methods (e.g. environmental DNA), calls for strong global commitments and collaborations to detect and close potential research and data gaps.

Threats to biodiversity are often connected to and maintained by supply chains rooted in biodiversity-rich developing countries, with industries (e.g. agriculture, forestry) geared towards export into wealthy countries [39]. Target locations of biodiversity-implicating commodities are in particular located in the USA, Europe, and Japan, emphasizing the need for a global recognition of threats to biodiversity [39]. At the same time, a much stronger and fairer North-South transfer of biodiversity-related research and knowledge is required.

Second, our global analyses show that biodiversity-related topics are not considered in a balanced way which might have an impact on conservation efforts. Research on the genetic and ecosystem level is increasing, most likely due to advanced molecular-biological, remote-sensing and modelling techniques and methods [40]. Furthermore, research on sub-species level is biased towards domesticated and cultivated varieties [41], while research on the ecosystem level remains in its infancy [42]. Consequently, current conservation strategies are based on species information, although the uncertainty of species numbers constrains conservation efforts [8,9]. Indeed, to enhance conservation strategies, substantial efforts are needed to fill current species-level knowledge gaps [43]. For example, viruses, fungi and parasites are much less studied than plants and animals [24,44], as confirmed in the present study too. Parasites are at very high risk of co-extinction, yet they are rarely considered in biodiversity research and conservation [44,45]. Furthermore, it may be ecosystem diversity rather than species diversity that primarily matters for the functioning of landscapes and entire biomes [46]. Indeed, to support effective conservation planning, *diversified* biodiversity-research efforts are needed, probably with ecosystems rather than species or genes as the ultimate target of management strategies.

The predominance of studies focusing on plants and vertebrates (Table 2), the uncertainties in global species numbers [47], and the focus on widespread and locally abundant species at the cost of small-ranging and rare species [48,49] point to a need to improve biodiversity research, as well as to extend monitoring programs on a global scale [1,41,50]. Here, paleoecological approaches to reconstruct biodiversity changes, and the underlying drivers, during the past decades to centuries, may strongly facilitate conservation efforts [51].

A strong conservation focus on terrestrial systems, as emphasized by Strayer (2006) too, might be rooted in the predominance of terrestrial biodiversity studies [52]. In contrast, marine and freshwater realms are under-represented in both research and protection [1]. In particular, a limited understanding of the conservation status of marine species, as well as their low detection abilities, may have caused an underestimation of extinction rates in the world's oceans [1,53]. Similarly, freshwater systems are among the most diverse and threatened systems globally. At the same time, they are highly underrepresented in biodiversity research and conservation planning [54].

The implementation of protected areas is considered a major step forward in successful conservation strategies [50], although it cannot be considered *per se* as an effective measure in reducing biodiversity loss [41]. Globally, the proportion of protected areas is increasing [55], and we identified a positive correlation between the proportion of protected areas and respective research activities. This correlation was even stronger than the correlations between the proportion of protected areas and indicators of actual biodiversity (number of ecoregions or number of threatened species). This suggests relatively poor guidance of conservation efforts by actual biodiversity data on a global scale. Also–and more important for our analyses–it suggests that conservation may indeed be a *consequence* of research activity rather than conservation and research being both dependent on existing biodiversity. Indeed, efficient conservation strategies may be hampered by the spatial disagreement between research subject and efforts, or in other words: The fact that biodiversity is not investigated where it is located has obvious implications for conservation planning.

Based on the observed spatial and topical imbalances in biodiversity-related research, we need fair collaborations across geopolitical borders. Comprehensive online data bases are needed to achieve ambitious goals such as the Aichi biodiversity targets. Halting the loss of biodiversity is a global challenge requiring spatially integrated and topically inclusive approaches, producing comprehensive and unbiased knowledge bases. Biodiversity is a resource that is exploited across geopolitical borders. Hence, policy makers–together with the scientific community–need to ensure cross-boundary research activities, including a global dissemination of data and knowledge. Care should be taken that research networks do not promote a new level of exploitation of biodiversity-rich and economically weak countries. Data base contributions should be mandatory, including open access to data bases and related biodiversity knowledge. Academic freedom is pivotal to avoid an exacerbated Matthew effect, to address new research and conservation directions, approaches and topics well beyond beaten paths.

## Supporting information

S1 Fig Comparison of automatic search (i.e. all publication titles and abstracts were automatically searched for search terms; Solid line) and subsample data (dashed line, with confidence interval) for research domain (terrestrial, freshwater, marine). (PDF)

S2 Fig Comparison of search algorithm (solid line) and subsample data (dashed line, with confidence interval) for level (genetic, phylogenetic, species, ecosystem). (PDF)

S3 Fig Comparison of search algorithm (solid line) and subsample data (dashed line, with confidence interval) for taxonomic group (plants and algae, vertebrates, invertebrates, bacteria, fungi, virus). (PDF)

S4 Fig Cartograms showing ratios between biodiversity-related research effort and biodiversity quantities. In **(A)** the size of each country represents the number of amphibian species (based on AmphibiaWeb); the color represents the ratio between publication count and number of amphibians. In **(B)** the size of each country represents the number of bird species (based on BirdLife). However, for bird species nearly no size change is observable; the color represents the ratio between publication count and number of bird species. In both (A) and (B) red countries have fewer studies per amphibian or per bird species and thus a relative biodiversity research deficit. The cartograms were generated using QGIS version 2.12.0 [20].
(PDF)

S1 Table Search terms for automatic search through publications. (XLSX)

S2 Table Core Biodiversity Scientists. (PDF)

S3 Table Correlation of number of ecoregions with number of amphibians and number of birds on country level (n = 201). (PDF)

S4 Table Comparison research effort considering all affiliations and only unique affiliations of first authors. (PDF)

S5 Table Institutions of biodiversity core scientists. Institutions with <5 core scientists are summarized to “other institutions”. (PDF)

S6 Table Categories of institutions of core scientists. (PDF)

S7 Table Gender distribution of core scientists in biodiversity research. (PDF)

S8 Table Correlation of GDP, number of threatened species, number of ecoregions and percentage of protected area with publications related to countries of authors’ affiliation **(A)** and countries of study site **(B)** (n = 199).
(PDF)
