# Supplementary material for: Spatial and topical imbalances in biodiversity research
Source: PLoS One. 2018 Jul 5;13(7):e0199327. doi: 10.1371/journal.pone.0199327 (PMC6033392; doi:10.1371/journal.pone.0199327)
Supplement: S1 Fig — (PDF) [file pone.0199327.s001.pdf]

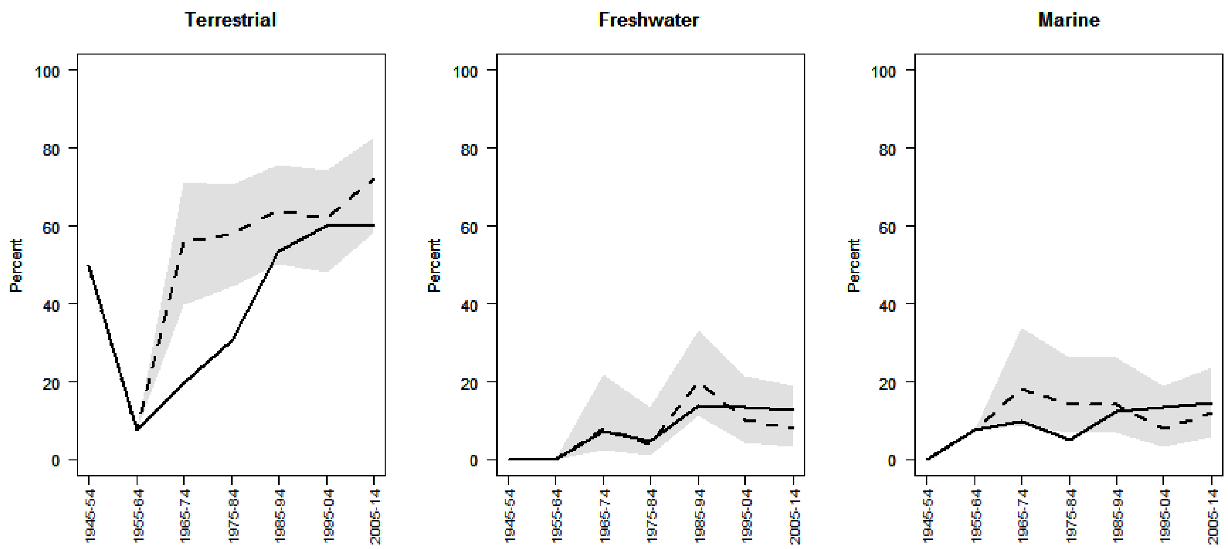

S1 Fig: Comparison of automatic search (i.e. all publication titles and abstracts were automatically searched for search terms; solid line) and subsample data (dashed line, with confidence interval) for research domain (terrestrial, freshwater, marine)
