# Supplementary material for: Spatial and topical imbalances in biodiversity research
Source: PLoS One. 2018 Jul 5;13(7):e0199327. doi: 10.1371/journal.pone.0199327 (PMC6033392; doi:10.1371/journal.pone.0199327)
Supplement: S2 Fig — (PDF) [file pone.0199327.s002.pdf]

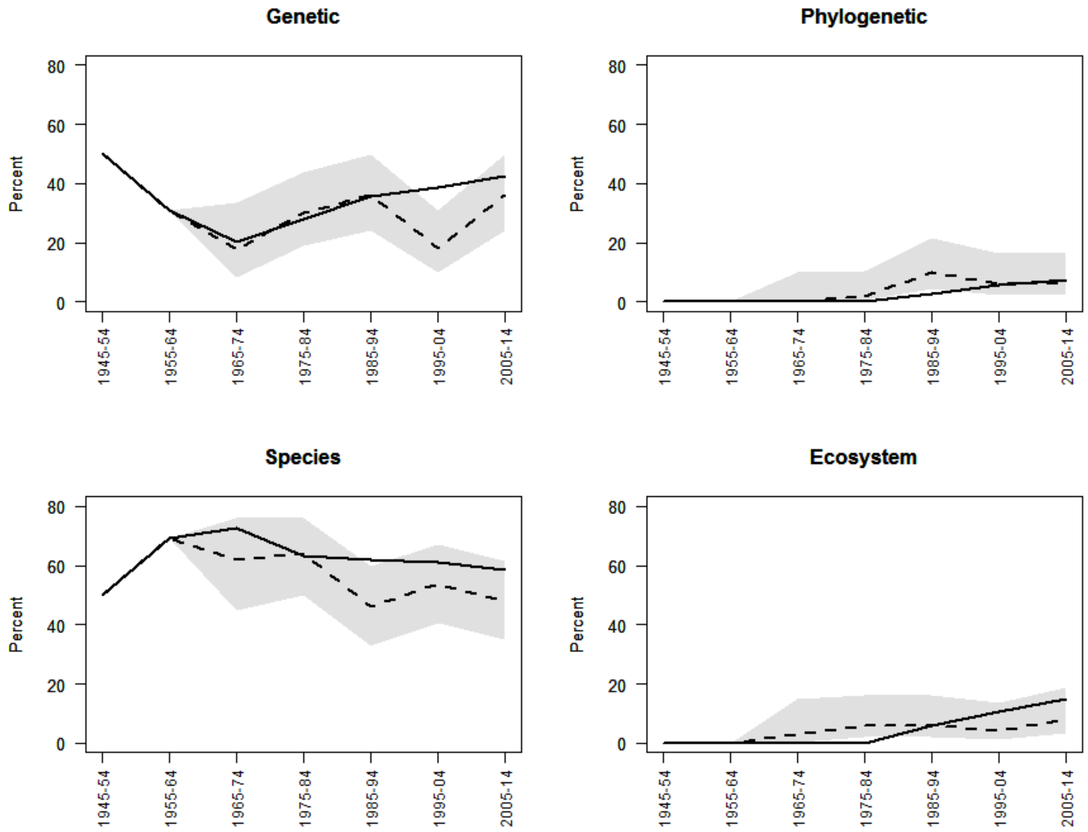

S2 Fig: Comparison of search algorithm (solid line) and subsample data (dashed line, with confidence interval) for level (genetic, phylogenetic, species, ecosystem).
