# Supplementary material for: Spatial and topical imbalances in biodiversity research
Source: PLoS One. 2018 Jul 5;13(7):e0199327. doi: 10.1371/journal.pone.0199327 (PMC6033392; doi:10.1371/journal.pone.0199327)
Supplement: S4 Fig — In (A) the size of each country represents the number of amphibian species (based on AmphibiaWeb); the color represents the ratio between publication count and number of amphibians. In (B) the size of each country represents the number of bird species (based on BirdLife). However, for bird species nearly no size change is observable; the color represents the ratio between publication count and number of bird species. In both (A) and (B) red countries have fewer studies per amphibian or per bird species and thus a relative biodiversity research deficit. The cartograms were generated using QGIS version 2.12.0 [20]. (PDF) [file pone.0199327.s004.pdf]

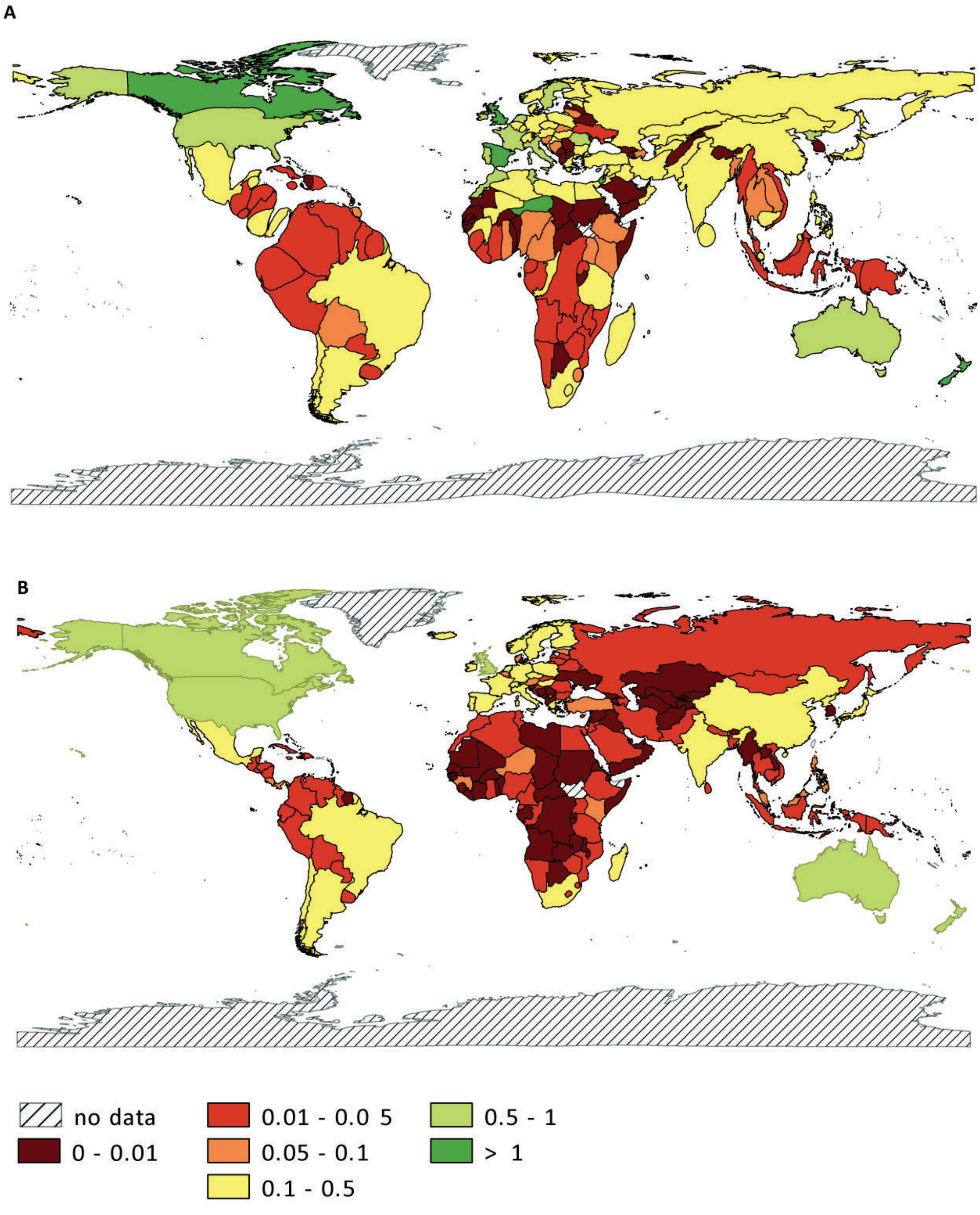

S4 Fig: Cartograms showing ratios between biodiversity-related research effort and biodiversity quantities. In **(A)** the size of each country represents the number of amphibian species (based on AmphibiaWeb); the color represents the ratio between publication count and number of amphibians. In **(B)** the size of each country represents the number of bird species (based on BirdLife). However, for bird species nearly no size change is observable; the color represents the ratio between publication count and number of bird species. In both (A) and (B) red countries have fewer studies per amphibian or per bird species and thus a relative biodiversity research deficit. The cartograms were generated using QGIS version 2.12.0 [20].
