# Supplementary material for: Spatial and topical imbalances in biodiversity research
Source: PLoS One. 2018 Jul 5;13(7):e0199327. doi: 10.1371/journal.pone.0199327 (PMC6033392; doi:10.1371/journal.pone.0199327)
Supplement: S2 Table — (PDF) [file pone.0199327.s006.pdf]

Table S2: Core Biodiversity Scientists. For each 5-year time period from 1945 to 2014, the twenty most frequently cited scientists, based on the total number of citations, were identified. Early time periods have fewer core scientists due to a lack of publications in these periods. In the list, the core scientists are generally ordered according to the number of citations for each time period (exceptions are researchers who are core scientists for several time periods; these are listed first).

Time period		Gender	Core Scientist	Institution	Country	Affiliation (PhD or current)	Number of publications (1945-2014)	Number of publications (current time period)		Times of citation (current time period)		Times of citation (1945-2014)	Betweenness Centrality (1945-2014)	Source			
1950 - 54			BLACK, GA				1	1		84		84	0		https://www.asas.org/docs/publications/england.pdf?sfvrsn=0	https://www.asas.org/docs/publications/england.pdf?sfvrsn=0	
1950 - 54		male	ENGLAND, DC	University of Minnesota<br>University of Minnesota	USA<br>USA	PhD<br>current	1	1		10		10	0	https://www.asas.org/docs/publications/england.pdf?sfvrsn=0	http://scarc.library.oregonstate.edu/findingaids/?p=collections/findingaid&id=75		
1960 - 64	1965-69	male	MACARTHUR, RH	Yale University<br>Princeton University	USA<br>USA	PhD<br>current	6	5	1	454		2256	0	http://uts.cc.utexas.edu/~varanus/MacArthur.html	https://en.wikipedia.org/wiki/Robert_MacArthur	http://www.oxfordbibliographies.com/view/document/obo-9780199830060/obo-9780199830060-	http://www.jstor.org/stable/2459142?seq=1#page_scan_tab_c
1960 - 64		male	LLOYD, M	 Oxford University	 UK	PhD<br>current	2	2		341		341	0	http://www.jstor.org/stable/2628?seq=1#page_scan_tab_contents	http://www.jstor.org/stable/2628?seq=1#page_scan_tab_contents		
1960 - 64		male	CONNELL, JH	University of Glasgow<br>University of California Santa Barbara	USA<br>USA	PhD<br>current	7	1		323		567	0	http://www.esa.org/history/Awards/bulletin/eminent1985.pdf	https://en.wikipedia.org/wiki/Joseph_H._Connell		
1960 - 64		male	KLOPFER, PH	Yale University<br>Duke University	USA<br>USA	PhD<br>current	3	2		184		189	0	http://dukespace.lib.duke.edu/dspace/handle/10161/877			
1960 - 64		male	PATTEN, BC	Rutgers University<br>University of Georgia	 USA	PhD<br>current	2	1		160		160	0	http://www.ecology.uga.edu/facultyMember.php?Patten-31			
1960 - 64		male	WALLACE, B	Columbia University<br>Cornell University	USA<br>USA	PhD<br>current	1	1		52		52	0	https://www.biol.vt.edu/OWLS/wallace_bio.html	http://www.nrcresearchpress.com/doi/abs/10.1139/g63-034?journalCode=cgc#.ViosJvkycVc		
1960 - 64		male	SUNESON, CA	Montana State University<br>University of California, Davis	USA<br>USA	PhD<br>current	1	1		47		47	0	http://texts.cdlib.org/view?docId=hb4q2nb2nd&doc.view=frames&chunk.id=div00066&toc.depth=1&toc.id=			
1965 - 69		male	PAINE, RT	University of Michigan<br>University of Washington	USA<br>USA	PhD<br>current	7	1		2713		2929	0	https://www.cornellcollege.edu/geology/courses/Greenstein/Paleoecology/readings/Paine1966.pdf			
1965 - 69		male	PIANKA, ER	University of Washington<br>University of Texas at Austin	USA<br>USA	PhD<br>current	11	2		1101		1453	0	https://integrativebio.utexas.edu/component/cobalt/item/7-integrative-biology/231-pianka-eric-	https://en.wikipedia.org/wiki/Eric_Pianka	http://biology.unm.edu/jhbrown/Documents/511Readings/Pianka1966.pdf	http://biology.unm.edu/jhbrown/Documents/511Readings/Pia
1965 - 69		female	PIELOU, EC	University of London<br>University of Lethbridge	UK<br>Canada	PhD<br>current	1	1		346		346	0	http://www.science.ca/scientists/scientistprofile.php?pID=208	http://www.esa.org/history/Awards/bulletin/eminent1986.pdf		
1965 - 69	1970-74	male	BUZAS, MA	Yale University<br>Smithsonian Institution, Washington, D.C.	USA<br>USA	PhD<br>current	12	1	1	282	110	743	0	http://paleobiology.si.edu/staff/individuals/buzas.html	https://www.sciencemag.org/content/163/3862/72.refs?related-urls=yes&legid=sci;163/3862/72		
1965 - 69	1970-74	male	GIBSON, TG	Princeton University<br>U. S. Geological Survey	USA<br>USA	PhD<br>current	4	1	1	282	110	400	0	http://www.cushmanfoundation.org/resources/researchergallery/gibson.php	https://books.google.de/books?id=a_FEiEm5CCQC&pg=PA82&lpg=PA82&dq=Thomas+G+Gibson+c		
1965 - 69		male	RECHER, HF	Stanford University<br>Edith Cowan University	USA<br>Australia	PhD<br>current	7	1		259		307	0	https://en.wikipedia.org/wiki/Robert_T._Paine_%28zoologist%29			
1965 - 69		male	STEHLI, FG	 Case Western Reserve University	 USA	PhD<br>current	2	2		226		226	0				
1965 - 69		male	SHELDON, AL	Cornell University<br>The University of Montana-Missoula	USA<br>USA	PhD<br>current	2	1		208		223	0	http://mus.edu/board/meetings/Archives/ITEM120-1008-R0703.htm			
1965 - 69		male	MONK, CD	Rutgers University<br>University of Georgia		PhD<br>current	4	3		109		148	0	http://www.plantbio.uga.edu/directory/carl-monk			
1965 - 69		male	SCHOENER, TW	Harvard University<br>University of California Davis	USA<br>USA	PhD<br>current	6	1		106		290	0	http://biosci3.ucdavis.edu/Faculty/Profile/View/244			
1965 - 69		male	HAIRSTON, NG	University of Washington<br>Cornell University	USA<br>USA	PhD<br>current	10	1		92		770	0	http://www.eeb.cornell.edu/hairston/CV.html			
1965 - 69		male	MOLLER, D	Justus Liebig University Gießen<br>University of Kassel	Germany	PhD<br>current	2	1		88		167	0	http://www.staff.uni-giessen.de/~gha5/	https://www.uni-kassel.de/fb11agrar/en/sections/department-of-		
1965 - 69			MACKAY, RJ				1	1		85		85	0				
1965 - 69		male	KOHN, AJ	Yale University<br>University of Washington	USA	PhD<br>current	3	1		83		206	0	http://www.biology.washington.edu/users/alan-j-kohn			
1965 - 69			WILHM, JL				2	1		80		89	0				
1965 - 69		male	JOHNSON, MP	Trinity College Dublin<br>Queen's College Galway	Ireland<br>Ireland	PhD<br>current	15	1		74		247	0	http://www.nuigalway.ie/our-research/people/natural-sciences/markjohnson/			
1965 - 69			WHITESID, MC				1	1		67		67	0				
1965 - 69			SLOBODKIN, LB				4	1		65		153	0	http://www.ncbi.nlm.nih.gov/pmc/articles/PMC2782105/			
1965 - 69		male	CAIRNS, J	University of Pennsylvania<br>Virginia Polytechnic Institute and State University	USA<br>USA	PhD<br>current	4	1		55		96	0	http://www.johncairns.net/Papers/cairnsvitae2015.pdf			
1970 - 74		male	HURLBERT, SH	Cornell University<br>San Diego State University	USA<br>USA	PhD<br>current	2	1		2015		2015	0	http://www.nwcouncil.org/media/14623/hurlbert.pdf	http://www.nwcouncil.org/media/14623/hurlbert.pdf		
1970 - 74		male	BROWN, JH	University of Michigan<br>The University of New Mexico	USA<br>USA	PhD<br>current	40	2		375		3177	0	http://www.webcitation.org/6RuGuoomo	http://www.nytimes.com/1999/01/12/science/of-mice-and-elephants-a-matter-of-scale.html?pagewanted=all&src=pm		
1970 - 74		male	LOYA, Y	State University of New York<br>Tel Aviv University	USA<br>Israel	PhD<br>current	3	1		314		750	0	http://www.tau.ac.il/lifesci/departments/zoology/members/loya/documents/FullCV.pdf			
1970 - 74		male	RAUP, DM	Harvard University<br>University of Chicago	USA<br>USA	PhD<br>current	3	1		296		646	0	http://news.uchicago.edu/article/2015/07/14/david-raup-paleontologist-who-transformed-his-discipline-	https://en.wikipedia.org/wiki/David_M._Raup		
1970 - 74		male	PORTER, JW	Yale University<br>University of Georgia	USA<br>USA	PhD<br>current	3	2		189		354	0	http://www.ecology.uga.edu/facultyMember.php?Porter-32/			
1970 - 74		male	BARBOUR, CD			PhD<br>current	1	1		152		152	0	http://www.mexfish.info/author_references.php?id=522&lang=de			
1970 - 74		male	ABELE, LG	University of Miami<br>Florida State University	USA<br>USA	PhD<br>current	2	2		128		189	0	https://fsu.edu/news/2010/09/17/provost.abele/	http://www.bio.fsu.edu/faculty-abele.php		
1970 - 74		male	SIMBERLOFF, DS	Harvard University<br>University of Tennessee	USA<br>USA	PhD<br>current	1	1		111		111	0	https://en.wikipedia.org/wiki/Daniel_Simberloff	http://www.icbi2013.org/admin/personfile/201302021750469987.pdf		
				University of Michigan	USA	PhD											

Time period	Gender	Core Scientist	Institution	Country	Affiliation (PhD or current)	Number of publications (1945-2014)	Number of publications (current time period)	Times of citation (current time period)	Times of citation (1945-2014)	Betweenness Centrality (1945-2014)	Source
1970 - 74	male	OWEN, DF	Oxford Brookes University	UK	current	6	5	110	133	0	http://self.gutenberg.org/articles/denis_owen#cite_note-independent1-2<br>http://www.independent.co.uk/news/obituaries/obituary-denis-owen-1359897.html
1970 - 74		BOROWITZ, MA			PhD	1	1	98	98	0	
1970 - 74		TOMOFF, CS				1	1	86	86	0	
1970 - 74	male	REX, MA	Harvard University<br>University of Massachusetts Boston	USA<br>USA	PhD<br>current	25	1	82	1235	0	https://www.umb.edu/academics/csm/faculty_staff/michael_rex
1970 - 74	male	DIAMOND, JM	University of Cambridge<br>University of California, Los Angeles	USA<br>UK	PhD<br>current	6	1	81	257	0	http://www.geog.ucla.edu/people/jared-diamond
1970 - 74		SHAFI, MI				1	1	77	77	0	
1970 - 74		CRAMER, NF				1	1	75	75	0	
1970 - 74		JARRY, B				1	1	73	73	0	
1970 - 74	female	OWEN, J				3	2	71	84	0	https://en.wikipedia.org/wiki/Denis_Owen
1970 - 74 1975 - 79	male	STRONG, DR	University of Oregon<br>University of California, Davis	USA<br>USA	PhD<br>current	6	1 4	67 231	343	0	http://strong.ucdavis.edu/files/don_bio.pdf<br>http://bml.ucdavis.edu/research/faculty/don-strong/
1975 - 79	male	GRUBB, PJ	University of Cambridge<br>University of Cambridge	UK<br>UK	PhD<br>current	6	1	2185	2427	0	https://en.wikipedia.org/wiki/Peter_J._Grubb<br>http://www.magd.cam.ac.uk/pjg12/<br>http://plants.jstor.org/stable/10.5555/al.ap.person.bm000046916
1975 - 79	male	HUSTON, M	University of Michigan<br>Texas State University	USA<br>USA	PhD<br>current	3	1	1827	2067	0	http://www.tsusinvasives.org/dotAsset/950331ff-372d-41c9-9133-71c0b0325fac
1975 - 79 1995-99	female	LUBCHENCO, J	Harvard University<br>Oregon State University	USA<br>USA	PhD<br>current	15	1 4	752	5755	198.79	http://gordon.science.oregonstate.edu/lubchenco/jlcv
1975 - 79	male	MENGE, BA	University of Washington<br>Oregon State University	USA<br>USA	PhD<br>current	21	1	747	1501	0	https://en.wikipedia.org/wiki/Bruce_A._Menge<br>http://gordon.science.oregonstate.edu/lubmengo2/?q=content/dr-bruce-allan-menge
1975 - 79	male	SOUSA, WP	 University of California, Berkeley	 USA	PhD<br>current	3	1	643	736	0	https://ib.berkeley.edu/people/faculty/sousaw
1975 - 79	male	HECK, KL	Florida State University<br>University of South Alabama	USA<br>USA	PhD<br>current	6	2	303	571	0	http://www.southalabama.edu/colleges/artsandsci/marinesciences/DrKenHeck.html<br>http://www.disl.org/faculty-staff/6
1975 - 79 1980-84 1985-89	male	NEVO, E	The Hebrew University of Jerusalem<br>University of Haifa	Israel<br>Israel	PhD<br>current	189	3 3 9	284 184 411	4562	0	http://evolution.haifa.ac.il/images/stories/Nevo_full_list_November_2013.pdf<br>https://en.wikipedia.org/wiki/Eviatar_Nevo<br>http://www.thethirdwayofevolution.com/people/view/eviatar-nevo
1975 - 79	male	JUMARS, PA	University of California, San Diego<br>University of Maine	USA<br>USA	PhD<br>current	3	2	256	294	0	http://www.whoi.edu/science/cicor/CVs/Jumars-CV_Maine.pdf
1975 - 79	male	BAZZAZ, FA	University of Illinois at Urbana-Champaign<br>Harvard University	USA<br>USA	PhD<br>current	13	1	255	865	0	http://www.planta.cn/forum/files_planta/fab_870.pdf
1975 - 79	male	ROTH, RR	 University of Delware	 USA	PhD<br>current	1	1	247	247	0	http://canr.udel.edu/our-faculty/enwc-faculty/
1975 - 79		WETSTONE, GS				1	1	243	243	0	
1975 - 79 1980-84	male	SEPKOSKI, JJ	Harvard University<br>University of Chicago	USA<br>USA	PhD<br>current	10	1 1	243 340	1365	148.0	https://en.wikipedia.org/wiki/Jack_Sepkoski
1975 - 79	male	TERBORGH, J	Harvard University<br>Duke University	USA<br>USA	PhD<br>current	10	1	240	1915	0	https://nicholas.duke.edu/people/faculty/terborgh<br>https://en.wikipedia.org/wiki/John_Terborgh
1975 - 79 1980-84 1985-89	male	SELANDER, RK	University of California, Berkeley<br>University of Rochester	USA<br>USA	PhD<br>current	17	2 3 6	236 558 736	2075	10.0	http://bio.psu.edu/news-and-events/2015-news/robert-selander-emeritus-professor-passes-away
1975 - 79		PARKER, ED				6	2	236	284	0	
1975 - 79		HABER, M				1	1	227	227	0	
1975 - 79	female	DAVIDSON, DW	 University of Utah	 USA	PhD<br>current	2	1	216	264	0	https://www.linkedin.com/pub/diane-davidson/67/713/a72<br>http://archive.unews.utah.edu/author/Diane%20%26%2338%3Bquot%3BDinah%26%2338%3Bquot%3B%20Davidson/
1975 - 79		CLARKE, BC				1	1	177	177	0	
1975 - 79	male	NILSSON, SG	 Lund University	 Sweden	PhD<br>current	17	4	166	886	0	http://www.biology.lu.se/sven-g-nilsson
1980 - 84	male	LEVIN, BR	 Emory University	 USA	PhD<br>current	3	2	521	637	0	http://www.eclf.net/home
1980 - 84 1985-89	male	SHMIDA, A	The Hebrew University of Jerusalem<br>The Hebrew University of Jerusalem	Israel<br>Israel	PhD<br>current	8	2 1	316 624	1064	0	http://www.bio.huji.ac.il/eng/staff_in.asp?staff_id=45&chapter_id=191
1980 - 84 1985-89	male	WILSON, MV	 Doane College, School of Arts and Sciences, Crete	 USA	PhD<br>current	8	1 1	313 624	985	148.0	http://www.doane.edu/mark-wilson
1980 - 84	male	HELTSHE, JF	 University of Rhode Island	 USA	PhD<br>current	2	1	310	336	0	http://www.cs.uri.edu/about-us/people/james-heltshe/
1980 - 84		FORRESTER, NE				1	1	310	310	0	

Time period	Gender	Core Scientist	Institution	Country	Affiliation (PhD or current)	Number of publications (1945-2014)	Number of publications (current time period)	Times of citation (current time period)	Times of citation (1945-2014)	Betweenness Centrality (1945-2014)	Source			
1980 - 84	male	SMITH, EP	University of Washington<br>Virginia Polytechnic Institute and State University	USA<br>USA	PhD<br>current	2	1	282	288	294.0	http://www.stat.vt.edu/people/faculty/Smith-Eric-CV.pdf			
1980 - 84	male	VANBELLE, G	University of Toronto<br>University of Washington	Canada<br>USA	PhD<br>current	1	1	282	282	0	http://sph.washington.edu/faculty/fac_bio.asp?url_ID=van_belle_gerald			
1980 - 84	male	TSUNEWAKI, K	 Fukui Prefectural University	 Japan	PhD<br>current	20	6	274	628	0	http://www.japan-acad.go.jp/en/members/6/tsunewaki_koichiro.html			
1980 - 84		OGIHARA, Y				8	3	243	553	0				
1980 - 84	female	DENSLOW, JS	University of Wisconsin Madison<br>USDA, Forest Service, Pacific Southwest Research Station	USA<br>USA	PhD<br>current	11	1	231	1030	0	http://www.fs.fed.us/psw/programs/ipif/staff/jdenslow/			
1980 - 84 1995-99	male	COLEMAN, BD	Yale University<br>Rutgers University	USA<br>USA	PhD<br>current	1	1	208 1947	208	0	http://www.mechanics.rutgers.edu/Vita_BDC_Jan08.pdf			
1980 - 84	female	CAUGANT, DA	 University of Oslo	 Norway	PhD<br>current	30	1	207	1498	0	https://www.med.uio.no/helsam/english/people/aca/dac/	http://www.fhi.no/eway/default.aspx?pid=240&trg=Content_6671&Main_6664=6898:0:25,7949:1:0:0:::0:0&MainContent_6898=6671:0:25,8029:1:0		
1980 - 84	male	GURIES, RP	University of Washington<br>University of Wisconsin	USA<br>USA	PhD<br>current	9	1	201	278	0	http://www.swchina.wisc.edu/cvs/cvguries.pdf			
1980 - 84	male	COPPOCK, DL	Colorado State University<br>Utah State University	USA<br>USA	PhD<br>current	3	1	176	202	0	https://qcnr.usu.edu/directory/coppock_layne			
1980 - 84	male	NIEMELA, P	 University of Turku	 Finland	PhD<br>current	9	4	165	284	0	https://www.researchgate.net/profile/Pekka_Niemelae			
1980 - 84	male	CHALFIE, M	Harvard University<br>Columbia University	USA<br>USA	PhD<br>current	1	1	149	149	0	http://www.columbia.edu/cu/biology/faculty/chalfie/			
1980 - 84	male	HASTINGS, JW	Princeton University<br>Harvard University	USA<br>USA	PhD<br>current	1	1	146	146	0	https://en.wikipedia.org/wiki/John_Woodland_Hastings	https://sleep.med.harvard.edu/people/faculty/211/In+memoriam+J+Woodland+Hastings+PhD		
1985 - 89	male	FRANKLIN, JF	Washington State University<br>University of Washington	USA<br>USA	PhD<br>current	14	1	497	1628	0	http://faculty.washington.edu/jff/index.htm			
1985 - 89	male	GENTRY, AH	Washington University<br>Missouri Botanical Garden	USA<br>USA	PhD<br>current	10	2	479	1512	0	http://www.jstor.org/stable/2399988?&seq=6#page_scan_tab_contents			
1985 - 89	male	MARGULES, CR	 James Cook University	 Australia	PhD<br>current	17	1	422	1738	148.0	https://research.jcu.edu.au/tess/people/staff/copy4_of_surname_initial-template			
1985 - 89 1990 - 94 2000-04	male	CURRIE, DJ	McGill University<br>University of Ottawa	Canada<br>Canada	PhD<br>current	39	1 3 15	410 862 2839	5105	0	http://mysite.science.uottawa.ca/dcurrie/index.html			
1985 - 89	male	KEDDY, PA	Dalhousie University<br>Southeastern Louisiana University	Canada<br>USA	PhD<br>current	18	3	256	980	0	http://www.drpaulkeddy.com/contact.html			
1985 - 89	male	WEISBURG, WG	University of Illinois at Urbana-Champaign<br>Scientific Affairs at Exact Sciences	USA<br>USA	PhD<br>current	1	1	243	243	0	https://www.linkedin.com/pub/will-weisburg/4/a9/210			
1985 - 89		DODSON, C				1	1	232	232	0				
1985 - 89	male	WENDEL, JF	University of North Carolina<br>Iowa State University	USA<br>USA	PhD<br>current	19	3	232	964	0	http://www.eeob.iastate.edu/faculty/WendelJ/pdfs/Wendel-cv-short.pdf			
1985 - 89	male	HAY, ME	University of California, Irvine<br>Georgia Institute of Technology	USA<br>USA	PhD<br>current	4	1	231	389	0	http://www.biology.gatech.edu/people/mark-hay	http://labs.biology.gatech.edu/labs/hay/		
1985 - 89	male	WILSON, EO	Harvard University<br>Harvard University	USA<br>USA	PhD<br>current	9	3	216	553	148.0	http://www.discoverlife.org/who/CV/Wilson,_Edward.html	https://en.wikipedia.org/wiki/E._O._Wilson	http://www.chgeharvard.org/about/people/edward-o-wilson	http://search.proquest.com/docview/301948222
1985 - 89 1990 - 94	male	GEPTS, P	University of Wisconsin<br>University of California, Davis	USA<br>USA	PhD<br>current	45	2 11	203 1082	2524	0	http://www.genetica.esalq.usp.br/29temas/cv/CV_Paul_Gepts.pdf			
1985 - 89	female	HALLOCK, P	University of Hawaii<br>University of South Florida	USA<br>USA	PhD<br>current	4	1	203	213	0	http://www.researchgate.net/profile/Pamela_Hallock	http://www.marine.usf.edu/reefslab/pmuller.html		
1985 - 89	male	QUINN, JF	 University of California, Davis	 USA	PhD<br>current	12	2	200	524	0	http://ice.ucdavis.edu/people/jfquinn			
1985 - 89	male	HEWLETT, EL	Johns Hopkins University<br>University of Virginia	USA<br>USA	PhD<br>current	1	1	193	193	0	http://www.fda.gov/downloads/AdvisoryCommittees/CommitteesMeetingMaterials/ScienceBoardtotheFoodandDrugAdministration/UCM245371.pdf			
			University of California Riverside	USA	PhD									

Time period				Gender	Core Scientist	Institution	Country	Affiliation (PhD or current)	Number of publications (1945-2014)	Number of publications (current time period)				Times of citation (current time period				Times of citation (1945-2014)	Betweenness Centrality (1945-2014)	Source			
1985 - 89	2000-04			male	HAWKINS, BA	University of California, Irvine	USA	current	84	1	18			183	4165			6875	365.12	http://www.faculty.uci.edu/profile.cfm?faculty_id=4562	http://www.faculty.uci.edu/profile.cfm?faculty_id=4562	http://www.faculty.uci.edu/profile.cfm?faculty_id=4562	
1985 - 89	1990-94	1995-99		male	LAWTON, JH	University of Durham<br>Imperial College London	UK<br>UK	PhD<br>current	42	3	9	19		180	2564	4367		8524	464.37	https://en.wikipedia.org/wiki/John_Lawton_%28biologist%29			
1990 - 94	1995-99			male	MUYZER, G	Leiden University<br>University of Amsterdam	Netherlands<br>Netherlands	PhD<br>current	33	1	10			5997	2389			9487	0	https://nl.linkedin.com/pub/gerard-muyzer/18/67b/64a	http://uchebana5.ru/cont/3982194.html	http://www.uva.nl/en/news-events/news/uva-news/content/professor-appointments/2011/11/dr-	
1990 - 94	1995-99	2000-04	2010-14	male	TILMAN, D	University of Michigan<br>University of California Santa Barbara	USA<br>USA	PhD<br>current	93	7	19	31	21	2887	6655	7351	1507	20853	384.69	http://www.bren.ucsb.edu/people/Faculty/david_tilman.htm	https://www.cbs.umn.edu/sites/default/files/public/downloads/2013,%20September,%20Tilman	http://www.sciencemag.org/content/192/4238/463.abstract?ijkey=06a46221fd216b73478e9098e67eb	
1990 - 94				male	WILLIAMS, PH	University of Cambridge<br>Natural History Museum London	UK<br>UK	PhD<br>current	43	4				1220				3725	181.99	http://www.nhm.ac.uk/our-science/departments-and-staff/staff-directory/paul-williams.html			
1990 - 94				male	NOSS, RF	University of Florida<br>University of Central Florida	USA<br>USA	PhD<br>current	26	4				1194				2108	0	http://biology.cos.ucf.edu/files/wp-content/uploads/2014/10/Reed-Noss-CV-Dec-2014.pdf	http://biology.cos.ucf.edu/faculty/reed-noss/	http://noss.cos.ucf.edu/	
1990 - 94				male	FAITH, DP	 Australian Museum Sydney	 Australia	PhD<br>current	31	3				1084				2492	770.42	http://australianmuseum.net.au/staff/dan-faith			
1990 - 94				male	DELONG, EF	University of California, San Diego<br>University of Hawaii	USA<br>USA	PhD<br>current	12	2				1013				2896	0	https://www.soest.hawaii.edu/oceanography/faculty/DeLong.html			
1990 - 94				male	GIOVANNONI, SJ	University of Oregon<br>Oregon State University	USA<br>USA	PhD<br>current	12	2				1008				2784	0	http://microbiology.science.oregonstate.edu/dr-stephen-giovannoni	http://www.bios.edu/about/team-members/stephen-giovannoni/		
1990 - 94				male	MELCHINGER, AE	University of Hohenheim<br>University of Hohenheim	Germany<br>Germany	current<br>PhD	71	15				970				2988	0	https://www.uni-hohenheim.de/person/albrecht-e-melchinger-27			
1990 - 94				female	LAWLER, SP	University of California, Davis<br>Rutgers University	USA<br>USA	current<br>PhD	8	2				928				1300	0	http://biosci3.ucdavis.edu/Faculty/Profile/View/14037			
1990 - 94				male	HUMPHRIES, CJ	Natural History Museum London<br>University of Reading	UK<br>UK	current<br>PhD	7	2				905				1200	37.55	https://chrisjh.wordpress.com/my-cv-2/			
1990 - 94	1995-99			male	NAEEM, S	University of California, Berkeley<br>Columbia University	USA<br>USA	PhD<br>current	35	1	10			884	1969			5300	238.63	http://www.columbia.edu/~sn2121/People/Naeem/Cv_full_3.pdf	http://earth.columbia.edu/articles/view/2580	http://www.columbia.edu/cu/e3b/faculty/naeem2.html	
1990 - 94				male	SKOLE, D	University of New Hampshire<br>Michigan State University	USA<br>USA	PhD<br>current	2	1				859				876	0	http://www.researchgate.net/profile/David_Skole/info	http://www.for.msu.edu/people/david_skole	http://www.goes.msu.edu/content.cfm?ID=14	
1990 - 94				male	VANEWRIGHT, RI	University of Copenhagen<br>University of Kent	Netherlands<br>UK	PhD<br>current	2	1				828				846	0	http://www.thethirdwayofevolution.com/people/view/richard-irwin-dick-vane-wright	https://www.kent.ac.uk/sac/staff-profiles/profiles/conservation-	http://www.ucl.ac.uk/taxome/jim/Mim/VaneW.html	
1990 - 94	1995-99			male	VITOUSEK, PM	Dartmouth College<br>Stanford University	USA<br>USA	PhD<br>current	6	2	3			803	3969			4772	0	http://web.stanford.edu/group/magma/vitousek509.htm	http://fsi.stanford.edu/people/Peter_Vitousek	https://woods.stanford.edu/about/woods-faculty/peter-vitousek	https://biology.stanford.edu/faculty \
1990 - 94				male	KNOLL, AH	Harvard University<br>Harvard University	USA<br>USA	PhD<br>current	9	2				799				1243	292.0	http://eps.harvard.edu/people/andrew-h-knoll			
1990 - 94				male	TOWNSEND, CR	 University of Otago	New Zealand 	PhD<br>current	34	3				749				2305	0	http://www.otago.ac.nz/zoology/staff/otago008936.html			
1990 - 94				male	EHRLICH, PR	University of Kansas<br>Stanford University	USA<br>USA	PhD<br>current	51	7				740				4175	314.79	https://woods.stanford.edu/about/woods-faculty/paul-ehrlich			
1995 - 99	2000-04			male	HEWITT, GM	University of Birmingham<br>University of East Anglia	UK<br>UK	PhD<br>current	14	4	5			3634	2551			6325	0	https://en.wikipedia.org/wiki/Godfrey_Hewitt	https://www.uea.ac.uk/biological-sciences/news/professor-godfrey-hewitt		
1995 - 99				male	MOONEY, HA	 Stanford University	 USA	PhD<br>current	20	3				3368				5172	786.09	http://fse.fsi.stanford.edu/people/harold_a_mooney			
1995 - 99				male	HAMRICK, JL	University of California, Berkeley<br>University of Georgia	USA<br>USA	PhD<br>current	96	35				2464				3973	0	https://www.plantbio.uga.edu/directory/james-l-hamrick			
1995 - 99	2000-04			male	LOREAU, M	Free University of Brussels<br>French National Centre for Scientific Research (CNRS) Moulis	Belgium<br>France	PhD<br>current	76	8	27			2389	4019			8008	122.097	http://www.researchgate.net/profile/Michel_Loreau/info	https://www.mcgill.ca/cambam/people/ecology-and-evolutionary-biological-group/loreau-michel		
1995 - 99				male	SIEMANN, E	University of Minnesota<br>Rice University	USA<br>USA	PhD<br>current	14	8				2237				2354	0	http://www.ruf.rice.edu/~siemann/evan.html			
1995 - 99					CORRELL, DL				2	2				2039				2039	0				
1995 - 99				male	CARPENTER, SR	University of Wisconsin-Madison<br>University of Wisconsin-Madison	USA<br>USA	PhD<br>current	9	3				1990				3025	835.68	http://limnology.wisc.edu/personnel/carpenter/Carpenter_FullCV_2012-11-23.pdf	http://www.siwi.org/prizes/stockholmwaterprize/laureates/stephen-r-carpenter-usa/	http://zoology.wisc.edu/faculty/car/car.html#research	
1995 - 99					WARD, JV				34	10				1929				4110	0				
1995 - 99				male	SMITH, VH	 University of Kansas	 USA	PhD<br>current	17	2				1918				2680	294.0	https://eeb.ku.edu/val-h-smith#link1			
1995 - 99				male	PETIT, RJ	University of Paris-South (Orsay)<br>INRA Head of the UMR Biogeco Research Unit, University Bordeaux	France<br>France	PhD<br>current	34	6				1886				5415	0	http://www6.bordeaux-aquitaine.inra.fr/biogeco_eng/Staff/Staff-directory/M-P/Petit-Remy			
1995 - 99				male	WIEMKEN, A	ETH Zürich<br>University of Basel	Switzerland<br>Switzerland	PhD<br>current	23	6				1697				3374	0	https://botanik.unibas.ch/personen/profil/person/wiemken/			
1995 - 99				male	BOLLER, T	ETH Zürich<br>University of Basel	Switzerland<br>Switzerland	PhD<br>current	14	5				1633				2838	0	https://botanik.unibas.ch/personen/profil/person/-b77335f368/			
1995 - 99				male	YACHI, S	Kyoto University<br>Kyoto University	Japan<br>Japan	PhD<br>current	5	2				1617				1682	0	http://www.ecology.kyoto-u.ac.jp/~yachi/index_E.html			
2000 - 04				male	DA FONSECA, GAB	University of Florida<br>Global Environment Facility	USA<br>USA	PhD<br>current	15	11				9896				10528	228.41	https://www.researchgate.net/profile/Gustavo_Fonseca4	https://www.thegef.org/gef/user/46		
2000 - 04				male	MITTERMEIER, RA	Harvard University<br>Conservation International	USA<br>USA	PhD<br>current	23	7				8136				9535	274.82	http://www.primate-sg.org/dr_russ_mittermeier/			
2000 - 04				male	PETERSON, AT	University of Chicago<br>University of Kansas	USA<br>USA	PhD<br>current	73	21				7644				10160	2.41	https://eeb.ku.edu/townsend-peterson#link1			

Time period	Gender	Core Scientist	Institution	Country	Affiliation (PhD or current)	Number of publications (1945-2014)	Number of publications (current time period)	Times of citation (current time period	Times of citation (1945-2014)	Betweenness Centrality (1945-2014)	Source
2000 - 04	male	MYERS, N	University of California, Berkeley<br>consultancy projects	USA 	PhD<br>current	10	2	7122	7717	436.55	https://fds.duke.edu/db/Nicholas/esp/faculty/normyers/files/CV.pdf
2000 - 04<br>2005-09	male	GASTON, KJ	University of York<br>University of Exeter	UK<br>UK	PhD<br>current	238	55<br>88	6479<br>3995	13276	526.99	http://kevingaston.com/biography/<br>http://www.exeter.ac.uk/esi/people/academicandhonorary/gaston/
2000 - 04	female	RODRIGUES, ASL	University of Sheffield<br>Centre National de la Recherche Scientifique (CNRS), Montpellier	UK<br>France	PhD<br>current	31	15	3471	4688	57.23	http://www.cefe.cnrs.fr/fr/recherche/bc/dpb/862-c/228-ana-rodrigues
2000 - 04	male	MITTELBACH, GG	Michigan State University<br>Michigan State University	USA<br>USA	PhD<br>current	22	12	3300	3683	199.28	http://www.kbs.msu.edu/people/faculty/mittelbach
2000 - 04<br>2005-09<br>2010-14	male	TSCHARNTKE, T	University of Hamburg<br>Georg-August-Universität Göttingen	Germany<br>Germany	PhD<br>current	172	22<br>58<br>83	2885<br>3643<br>1795	9452	423.3	https://www.uni-goettingen.de/de/92552.html
2000 - 04<br>2010-14	male	STUART, SN	University of Cambridge<br>IUCN Bath	UK<br>UK	PhD<br>current	23	6<br>11	2848<br>1254	4779	118.28	http://www.iucn.org/media/iucn_experts/?11636/Simon-Stuart-Chair-of-the-IUCN-Species-Survival-<br>https://uk.linkedin.com/pub/simon-stuart/8/a01/b74
2000 - 04<br>2010 - 14	male	SCHMID, B	University of Zurich<br>University of Zurich	Switzerland<br>Switzerland	PhD<br>current	144	35<br>59	2715<br>1127	6933	585.41	http://www.ieu.uzh.ch/en/staff/professors/bschmid.html#5
2000 - 04	male	PRESSEY, RL	University of New South Wales<br>James Cook University	Australia<br>Australia	PhD<br>current	86	27	2674	5411	336.93	https://www.researchgate.net/profile/Robert_Pressey/info
2000 - 04	male	STEFFAN-DEWENTER,	Georg-August-Universität Göttingen<br>University of Würzburg	Germany<br>Germany	PhD<br>current	89	21	2630	5516	0	http://www.zoo3.biozentrum.uni-wuerzburg.de/en/team/steffan_dewenter/#c272233
2000 - 04	male	PORTER, EE	 U.S. Fish and Wildlife Service	 USA	PhD<br>current	14	12	2560	2583	0	https://www.researchgate.net/profile/Eric_Porter
2000 - 04	male	BROOKS, TM	 IUCN	 USA	PhD<br>current	33	12	2523	4888	124.59	http://www.iucn.org/media/iucn_experts/?11667/Thomas-Brooks-Head-IUCN-Science-and-Knowledge-Unit
2000 - 04	male	CONDIT, R	University of California, Santa Crux<br>Smithsonian Tropical Research Institute	USA<br>Panama	PhD<br>current	38	16	2505	4459	0	https://www.stri.si.edu/english/scientific_staff/staff_scientist/scientist.php?id=5
2005 - 09	male	EXCOFFIER, L	University of Geneva<br>University of Berne	Switzerland<br>Switzerland	PhD<br>current	33	12	8988	10408	0	http://www.cmpg.iee.unibe.ch/content/about_us/researchers/laurent_excoffier/index_eng.html
2005 - 09<br>2010-14	male	THUILLER, W	Centre d'Ecologie Fonctionnelle et Evolutive, Univ. Montpellier I and Centre National de la Recherche Scientifique<br>CNRS Laboratoire d'Ecologie Alpine (LECA), Grenoble, France	France<br>France	PhD<br>current	106	41<br>57	5256<br>1627	8599	60.84	http://www.will.chez-alice.fr/About_me.html
2005 - 09	male	SCHLOSS, PD	Cornell University<br>University of Michigan medical School	USA<br>USA	PhD<br>current	15	10	4870	5182	432.0	http://www.med.umich.edu/MICROBIO/bio/schloss.htm
2005 - 09	male	EVANNO, G	University of Lausanne<br>INRA	Switzerland<br>France	PhD<br>current	2	2	4794	4794	0	http://www6.rennes.inra.fr/ese_eng/PEOPLE/Personne_umr/%28idpers%29/216/%28idlang%29/uk<br>http://www.unil.ch/dee/en/home/menuinst/people/previous-collaborators/guillaume-
2005 - 09	male	PHILLIPS, SJ	 AT&T Labs-Research	 USA	PhD<br>current	9	7	4638	4676	22.19	http://apps.webofknowledge.com/full_record.do?product=WOS&search_mode=DaisyOneClickSearch&qid=46&SID=P1zhyppMrEfRsEwldtz&page=2&doc=13
2005 - 09	male	ARAUJO, MB	University College London<br>Spanish Research Council	UK<br>Spain	PhD<br>current	73	31	4406	7328	483.55	http://www.maraujolab.com/people/miguel-araujo/
2005 - 09	male	HALPERN, BS	University of California, Santa Barbara<br>UC Santa Barbara<br>Imperial College London	USA<br>USA<br>UK	PhD<br>current<br>current2	27	12	4011	4496	151.93	http://www.bren.ucsb.edu/people/faculty/ben_halpern.htm<br>http://msi.ucsb.edu/people/faculty/benjamin-halpern<br>http://www.imperial.ac.uk/people/b.halpern<br>http://benhalpernlab.org/
2005 - 09	male	FOLKE, C	Stockholm University<br>Stockholm Resilience Centre	Sweden<br>Sweden	PhD<br>current	31	11	3663	6410	87.28	http://www.stockholmresilience.org/21/contact/staff/1-15-2008-folke.html<br>https://en.wikipedia.org/wiki/Carl_Folke_%28ecologist%29
2005 - 09<br>2010-14	male	POSSINGHAM, HP	Oxford University<br>University of Queensland	UK<br>Australia	PhD<br>current	175	59<br>96	3546<br>1231	6066	1065.49	http://www.uq.edu.au/spatialecology/docs/Possingham-CV-Jan-2012.pdf
2005 - 09	male	WATSON, R	University of Queensland<br>University of Tasmania	Australia<br>Australia	PhD<br>current	20	4	3094	4649	310.04	http://www.utas.edu.au/profiles/staff/imas/reg-watson
2005 - 09	female	MICHELI, F	University of North Carolina<br>Stanford University	USA<br>USA	PhD<br>current	33	11	2974	3716	33.39	http://micheli.stanford.edu/micheli.html<br>http://micheli.stanford.edu/pdf/MicheliCV.pdf
2005 - 09	male	HEBERT, PDN	University of Cambridge<br>University of Guelph	UK<br>Canada	PhD<br>current	103	28	2863	5909	0	http://www.uoguelph.ca/ib/people/faculty/hebert.shtml
			University of Kiel	Germany	PhD						http://www.dal.ca/faculty/science/biology/faculty-<br>http://wormlab.biology.dal.ca/about/who-are-<br>http://www.dfg.de/service/presse/pressemitteilun

Time period		Gender	Core Scientist	Institution	Country	Affiliation (PhD or current)	Number of publications (1945-2014)	Number of publications (current time period)		Times of citation (current time period		Times of citation (1945-2014)	Betweenness Centrality (1945-2014)	Source		
2005 - 09		male	WORM, B	Dalhousie University	Canada	current	28	11		2745		3966	25.13	http://www.dal.ca/faculty/science/biology/faculty-staff/our-faculty/boris-worm/boris-worm.html	http://wormlab.biology.dal.ca/about/who-are-we/dr-boris-worm/	http://www.dfg.de/service/presse/pressemitteilun[illegible]gen/2004/pressemitteilung_nr_12/index.html
2005 - 09	2010-14	female	LAVOREL, S	 CNRS Laboratoire d'Ecologie Alpine (LECA), Grenoble, France	 France	PhD<br>current	78	19	35	2743	1123	5701	108.96	http://www.ae-info.org/ae/User/Lavorel_Sandra		
2005 - 09		male	HARTMANN, M	ETH Zurich<br>Swiss Federal Institute for Forest, Snow and Landscape Research	Switzerland<br>Switzerland	PhD<br>current	6	1		2733		2771	0	http://www.wsl.ch/info/mitarbeitende/hartmann/index_EN		
2005 - 09		male	VAN HORN, DJ	 The University of New Mexico	 USA	PhD<br>current	8	2		2670		2685	0	https://www.researchgate.net/profile/David_Van_Horn/publications		
2005 - 09		male	PARKS, DH	 University of Queensland	 Australia	PhD<br>current	5	2		2661		2670	0	http://dparks.wikidot.com/start		
2005 - 09		male	DUFFY, JE	University of North Carolina<br>The College of William and Mary	USA<br>USA	PhD<br>current	43	19		2639		3914	141.08	http://www.vims.edu/people/duffy_je/cv/Duffy_CV_Jan_2013.pdf	http://www.vims.edu/people/duffy_je/	
2010 - 14		male	KNIGHT, R	Princeton University<br>University of Colorado, Boulder	USA<br>USA	PhD<br>current	42	33		1659		4161	568.0	http://chem.colorado.edu/index.php?option=com_content&view=article&id=263:rob-		
2010 - 14		male	BUTCHART, SHM	 Cambridge Conservation Initiative Cambridge Judge Business School / BirdLife International<br>IUCN SSC Climate Change Specialist Group	 UK 	PhD<br>current<br>current2	46	34		1522		2456	264.09	https://iucn-ccsg.org/people/stuart-butchart	http://www.conservation.cam.ac.uk/person/dr-stuart-butchart	
2010 - 14		male	COLLEN, B	Imperial College London<br>University College London	UK<br>UK	PhD<br>current	34	24		1382		2449	185.99	http://www.collenlab.org/ben-collen.html		
2010 - 14		male	BAILLIE, JEM	 Zoological Society of London	 UK	PhD<br>current	21	12		1292		2049	32.99	https://www.researchgate.net/profile/Jonathan_Baillie/publications		
2010 - 14		male	CARPENTER, KE	University of Hawaii<br>Old Dominion University	USA<br>USA	PhD<br>current	24	22		1268		1391	835.68	http://sci.odu.edu/biology/directory/kent.shtml		
2010 - 14		male	REICH, PB	Cornell University<br>University of Minnesota	USA<br>USA	PhD<br>current	84	39		1227		4884	206.01	https://forestry.umn.edu/sites/forestry.umn.edu/files/pbrfullcvdec2015.pdf	https://www.forestry.umn.edu/people/peter-b-reich	
2010 - 14		male	WEISSER, WW	Oxford University<br>Technische Universität München	UK<br>Germany	PhD<br>current	98	73		1184		2499	19.1	http://www.toek.wzw.tum.de/index.php?id=69		
2010 - 14		male	SCHARLEMANN, JPW	University of Cambridge<br>University of Sussex	UK<br>UK	PhD<br>current	23	17		1084		1933	292.57	http://www.sussex.ac.uk/profiles/306025		
2010 - 14		male	WATSON, R	University of Queensland<br>University of Tasmania	Australia<br>Australia	PhD<br>current	20	13		1079		4649	310.04	http://www.utas.edu.au/profiles/staff/imas/reg-watson		
2010 - 14		male	ACKERLY, DD	 University of California, Berkeley	 USA	PhD<br>current	17	12		1036		2094	75.1	https://ib.berkeley.edu/people/faculty/ackerlyd		
2010 - 14		male	TEDERSOO, L	University of Montpellier<br>University of Tartu	France<br>Estonia	PhD<br>current	22	16		1030		1379	0	https://www.etis.ee/Portal/Persons/Display/5bdd93e8-9546-4b4b-b5e6-ee5d7d0349f0?tabId=CV_ENG		
2010 - 14		male	GENOVESI, P	University of Rome<br>Institute for Environmental Protection and Research (ISPRA)	Italy<br>Italy	PhD<br>current	13	11		1021		1209	52.27	http://www.issg.org/contact.htm	https://www.researchgate.net/profile/Piero_Genovesi/info	
2010 - 14		male	MOUILLOT, D	 University of Montpellier	 France	PhD<br>current	109	48		1012		3141	49.49	http://www.umr-marbec.fr/mouillot-david.html?lang=fr		

Sensitivity analysis

Time period		Gender	Core Scientist	Institution	Country	Affiliation (PhD or current)	Number of publications (1945-2014)	Number of publications (current time period)		Times of citation (current time period		Times of citation (1945-2014)	Betweenness Centrality (1945-2014)	Source	
1980 - 84		male	ZOHARY, D	University of California, Berkeley<br>University of California, Berkeley	USA<br>USA	PhD<br>current	8	1		143		616	-	http://www.danielzohary.com/Home/publications	
1980 - 84		male	BROWN, AHD	University of California, Davis<br>Centre for Plant Biodiversity Research Australia	USA<br>Australia	PhD<br>current	12	1		143		1078	-	https://www.anbg.gov.au/cpbr/program/sc/brown_staff.htm	
1980 - 84		male	BEILES, A	Hebrew University of Jerusalem<br>Hebrew University, Jerusalem	Israel<br>Israel	PhD<br>current	37	1		143		1442	-	http://evolution.haifa.ac.il/index.php/27-people/cv/72-cv-a-beiles	
1980 - 84		male	RICHERSON, PJ	University of California, Davis<br>University of California Davis	USA<br>USA	PhD<br>current	2	1		142		165	-	http://www.des.ucdavis.edu/faculty/Richerson/Richerson.htm	
1980 - 84	1975 - 79	male	HUSTON, M	University of Michigan<br>Texas State University	USA<br>USA	PhD<br>current	3	1	1	131	1827	2067	-	http://www.tsusinvasives.org/dotAsset/950331ff-372d-41c9-9133-71c0b0325fac	
1980 - 84		male	GREENSTONE, MH	University of California , Berkeley<br>United States Department of Agriculture	USA<br>USA	PhD<br>current	1	1		130		130	-	http://www.ars.usda.gov/pandp/people/people.htm?personid=33981	
1980 - 84		male	VERMEER, JG	University of Utrecht<br>Wageningen UR	Netherlands<br>Netherlands	PhD<br>current	1	1		126		126	-	http://link.springer.com/article/10.1007/BF00043032#page-1	
1980 - 84		male	BERENDSE, F	Rijksuniversiteit Utrecht<br>Universiteit Wageningen	Netherlands<br>Netherlands	PhD<br>current	44	1		126		2181	-	http://www.natuurinnederland.nl/NiN_auteur.html	
1980 - 84		male	MORAN, GF	 CSIRO	 Australia	PhD<br>current	23	1		124		848	-	http://www.genetics.org/content/171/3/1257.article-info	
1980 - 84		male	HOPPER, SD	The University of Western Australia<br>The University of Western Australia	Australia<br>Australia	PhD<br>current	5	1		124		223	-	http://www.web.uwa.edu.au/profile?dn=cn%253DStephen%2520Hopper%252Cou%253DCentre%2520of%2520Excellence%2520in%2520Natural%2520Resource%2520Management%252Cou%253DFaculty%2520of%2520Science%252Cou%253DFaculties%252Co%253DThe%2520University%2520of%2520Western%2520Australia	
2010 - 14		male	KUHN, I	University of Bochum<br>Martin-Luther-University Halle-Wittenberg	Germany<br>Germany	PhD<br>current	60	35		1004		2733	-	http://www.ufz.de/index.php?en=38592#Curriculum%20Vitae	
2010 - 14		male	QUADER, S	University of Florida<br>Nature Conservation Foundation	USA<br>India	PhD<br>current	5	4		1001		1059	-	https://scholar.google.de/citations?user=xSHih2sAAAAJ&hl=de	http://ncf-india.org/people/suhel-quader
2010 - 14		female	CHENERY, AM	 United Nations Environment Programme World Con	 UK	PhD<br>current	4	4		989		989	-	http://www.sciencemag.org/author/anna-m-chenery	
2010 - 14		male	SYMES, A	 BirdLife International	 UK	PhD<br>current	4	16		988		988	-	https://www.researchgate.net/profile/Andy_Symes	
2010 - 14		male	BOMMARCO, R	 Swedish University of Agricultural Sciences	 Sweden	PhD<br>current	44	37		974		2140	-	http://www.slu.se/ecology/riccardobommarco	
2010 - 14		female	MCGEOCH, MA	Pretoria University<br>Monash University Australia	South Africa<br>Australia	PhD<br>current	40	18		951		1530	-	https://www.monash.edu/research/people/profiles/profile.html?sid=2951597&pid=7041	
2010 - 14		male	JETZ, W	University of Oxford<br>Yale University	UK<br>USA	PhD<br>current	51	26		939		3296	-	http://jetzlab.yale.edu/people/walter-jetz	
2010 - 14		male	PAULY, D	University of Kiel	Germany	PhD	18	9		929		2274	-	http://fisheries.sites.olt.ubc.ca/files/2015/07/DPCV.pdf	

Time period	Gender	Core Scientist	Institution	Country	Affiliation (PhD or current)	Number of publications (1945-2014)	Number of publications (current time period)	Times of citation (current time period	Times of citation (1945-2014)	Betweenness Centrality (1945-2014)	Source
[illegible]	[illegible]	[illegible]	University of British Columbia	USA	current	[illegible]	[illegible]	[illegible]	[illegible]		[illegible]
2010 - 14	male	KOLJALG, U	University of Helsinki	Finland	PhD	17	11	897	1354	-	http://www.botany.ut.ee/urmas.koljalg/cv.html
			University of Tartu	Finland	current						
2010 - 14	male	BALZER, C			PhD	2	2	888	888	-	http://www.msi.ucsb.edu/luce-fellowship/recipients/christian-balzer
			University of California, Santa Barbara	USA	current						
