# Supplementary material for: Spatial and topical imbalances in biodiversity research
Source: PLoS One. 2018 Jul 5;13(7):e0199327. doi: 10.1371/journal.pone.0199327 (PMC6033392; doi:10.1371/journal.pone.0199327)
Supplement: S3 Table — (PDF) [file pone.0199327.s007.pdf]

S3 Table: Correlation of number of ecoregions with number of amphibians and number of birds on country level (n = 201)

	**correlation coefficient**	**p-value**
**Amphibians**	0.75	< 0.001
**Birds**	0.81	< 0.001
