# Supplementary material for: Spatial and topical imbalances in biodiversity research
Source: PLoS One. 2018 Jul 5;13(7):e0199327. doi: 10.1371/journal.pone.0199327 (PMC6033392; doi:10.1371/journal.pone.0199327)
Supplement: S4 Table — (PDF) [file pone.0199327.s008.pdf]

S4 Table: Comparison research effort considering all affiliations and only unique affiliations of first authors.

	**Research effort considering all author affiliations (%)**	**Research effort considering first-author affiliations (%)**
**Africa**	6.45	2.87
**Asia**	18.61	16.65
**Europe**	31.24	24.36
**North America**	23.21	16.56
**South America**	8.36	7.37
**Central America**	3.41	1.66
**Caribbean**	0.35	0.06
**Oceania**	8.09	5.58
