# Supplementary material for: Spatial and topical imbalances in biodiversity research
Source: PLoS One. 2018 Jul 5;13(7):e0199327. doi: 10.1371/journal.pone.0199327 (PMC6033392; doi:10.1371/journal.pone.0199327)
Supplement: S5 Table — (PDF) [file pone.0199327.s009.pdf]

S5 Table: Institutions of biodiversity core scientists. Institutions with <5 core scientists are summarized to "other institutions".

Institution	PhD affiliation	Current affiliation	Total
Harvard University	11	4	15
University of Washington	5	3	8
University of Cambridge	6	1	7
Cornell University	4	2	6
University of California, Davis	-	6	6
University of Michigan	5	1	6
Yale University	6	-	6
Stanford University	1	4	5
University of California, Berkeley	4	1	5
University of Wisconsin	3	2	5
Other institutions	78	116	194
