# Supplementary material for: Spatial and topical imbalances in biodiversity research
Source: PLoS One. 2018 Jul 5;13(7):e0199327. doi: 10.1371/journal.pone.0199327 (PMC6033392; doi:10.1371/journal.pone.0199327)
Supplement: S6 Table — (PDF) [file pone.0199327.s010.pdf]

S6 Table: Categories of institutions of core scientists

Governmental institutions	Non profit and private organizations	Private enterprise	Research Institutions and museums	Universities and Colleges
6	7	3	14	241
