# Supplementary material for: Spatial and topical imbalances in biodiversity research
Source: PLoS One. 2018 Jul 5;13(7):e0199327. doi: 10.1371/journal.pone.0199327 (PMC6033392; doi:10.1371/journal.pone.0199327)
Supplement: S7 Table — (PDF) [file pone.0199327.s011.pdf]

S7 Table: Gender distribution of core scientists in biodiversity research.

Decade	Number of female scientists	Number of male scientists
1945 - 54	0	1
1955 - 64	0	7
1965 - 74	1	26
1975 - 84	4	26
1985 - 94	2	30
1995 - 04	1	26
2005 - 14	2	28
**Total**	**10**	**144**
