# Supplementary material for: Spatial and topical imbalances in biodiversity research
Source: PLoS One. 2018 Jul 5;13(7):e0199327. doi: 10.1371/journal.pone.0199327 (PMC6033392; doi:10.1371/journal.pone.0199327)
Supplement: S8 Table — Correlation of GDP, number of threatened species, number of ecoregions and percentage of protected area with publications related to countries of authors’ affiliation (A) and countries of study site (B) (n = 199). (PDF) [file pone.0199327.s012.pdf]

S8 Table: Correlation of GDP, number of threatened species, number of ecoregions and percentage of protected area with publications related to countries of authors' affiliation **(A)** and countries of study site **(B)** (n = 199)

**A**	**correlation coefficient**	**p-value**
GDP	0.86	< 0.001
Threatened species	0.49	< 0.001
Ecoregions	0.35	2.341e-07
Protected area	0.51	5.551e-15

**B**	**correlation coefficient**	**p-value**
GDP	0.67	< 0.001
Threatened species	0.61	< 0.001
Ecoregions	0.61	< 0.001
Protected area	0.77	< 0.001
